# Strategies towards designing visible and near-infrared light driven molecular motors

**DOI:** 10.1039/d5cs00869g

**Published:** 2026-10-19

**Authors:** Yuyang Tu, Wojciech Danowski, Charlotte N. Stindt, Jinyu Sheng, Ben L. Feringa

**Affiliations:** a Suzhou Dushu Lake Hospital, Center of Translational Medicine, The Fourth Affiliated Hospital of Soochow University, Medical Center of Soochow University & State Key Laboratory of Bioinspired Interfacial Materials Science & Innovation Center for Advanced Polymer Materials & College of Chemistry, Chemical Engineering and Materials Science, Soochow University Suzhou Jiangsu 215123 China jysheng@suda.edu.cn; b Stratingh Institute for Chemistry, University of Groningen Groningen 9747 AG The Netherlands b.l.feringa@rug.nl; c Faculty of Chemistry, University of Warsaw Warsaw 02-093 Poland

## Abstract

Light is one of the most powerful and versatile stimuli for controlling molecular motion, serving as a key driving force in the development of photoresponsive molecular systems. Among these architectures, artificial molecular motors represent a unique class of molecules capable of converting light energy into chirality-controlled unidirectional rotary motion. However, the majority of reported examples are operated under ultraviolet excitation, which restricts their practical applicability especially in biomolecular systems and sensitive responsive materials. To address these limitations, major recent effort has been devoted to extending photoresponsiveness of molecular motors into the visible and near-infrared (NIR) regions which enables milder and more efficient operation. This review summarizes the principal molecular design strategies that have been developed to achieve such red-shifted photoactivation of molecular motors. Moreover, we highlight the current challenges and outline future directions toward the realization of high-efficiency, multifunctional, and programmable visible/NIR-light-driven molecular motors for integration into responsive materials, catalysis, and biological systems.

## Abstract 2

This review summarizes molecular design strategies for shifting molecular motors from UV to visible and near-infrared operation, with emphasis on recent advances and emerging applications.

## Graphical abstract

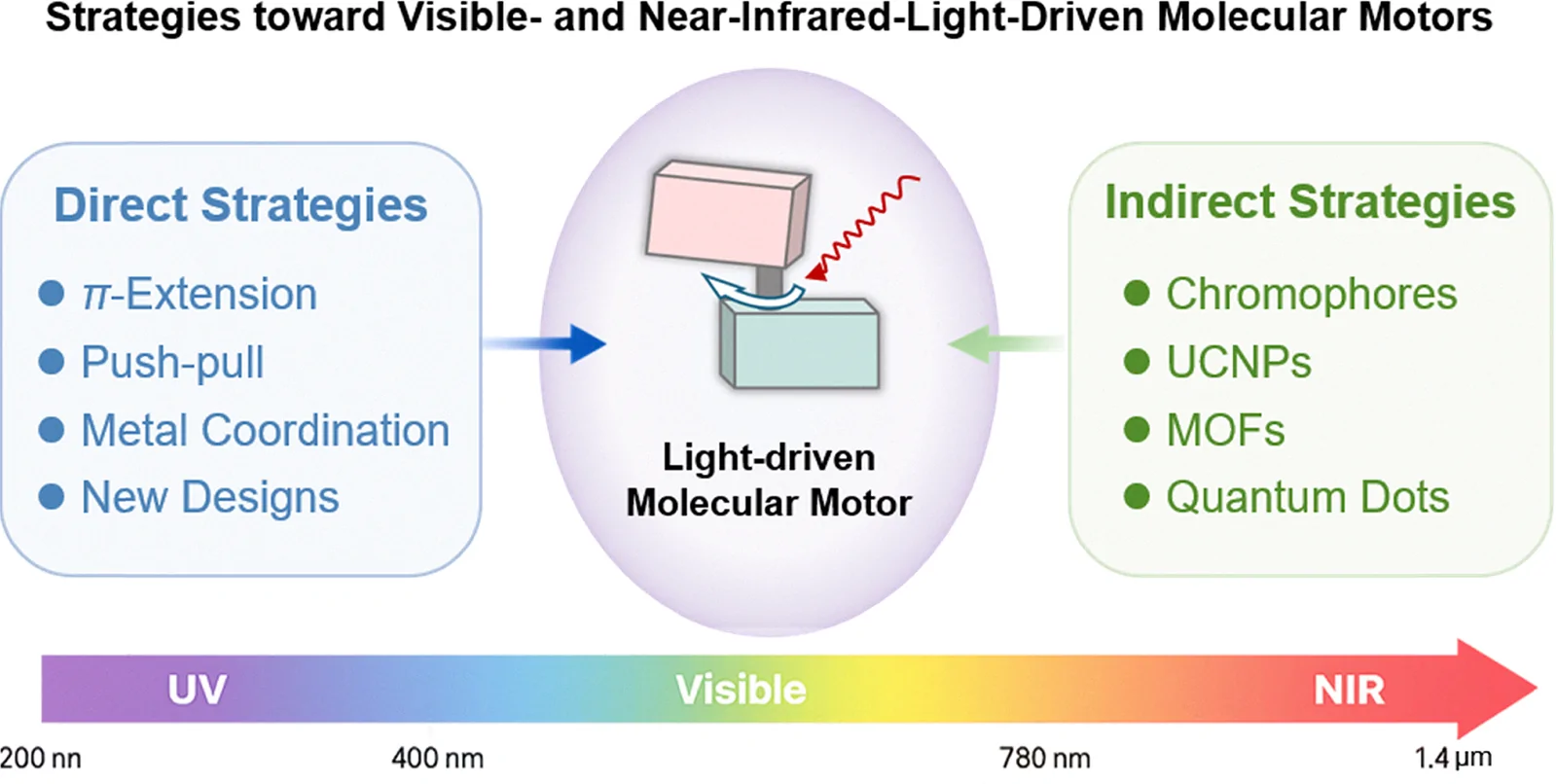

**Key learning points**

1. The importance and necessities of red-shifting photoactivation from UV to visible and near-infrared regions.

2. Design principles and major molecular strategies for visible/NIR-light-driven motors.

3. Key challenges in motor development and directions towards responsive systems.

4. The future potential of visible/NIR-driven molecular motors.

## 1. Introduction

Light constitutes a primary source of low-entropy energy flux in nature. From photosynthesis powering the biosphere, to the process of vision and light-dependent DNA repair, safeguarding genetic material from UV-induced damage and translating light into chemical and electrical signals, biological systems have evolved exquisite molecular machinery. These systems convert photons into mechanical or chemical work with remarkable spatiotemporal precision. Phototransduction in vision relies on an ultrafast *cis–trans* photoisomerisation of the retinal chromophore confined in the protein pocket, which triggers a cascade of conformational changes that ultimately generate a nerve impulse (Fig. 1a, left).^1^ Likewise, key photochemical events occur in photosynthesis and DNA photoreactivation, where molecular structure directs the energy flow and governs the photochemical selectivity.^2,3^ These evolutionarily optimised systems represent a benchmark for effective light utilisation, demonstrating that precisely controlled, light-induced conformational changes at the molecular scale—often referred to as molecular motion—enable the conversion of absorbed energy into chemical and possibly mechanical work.

**Fig. 1:**
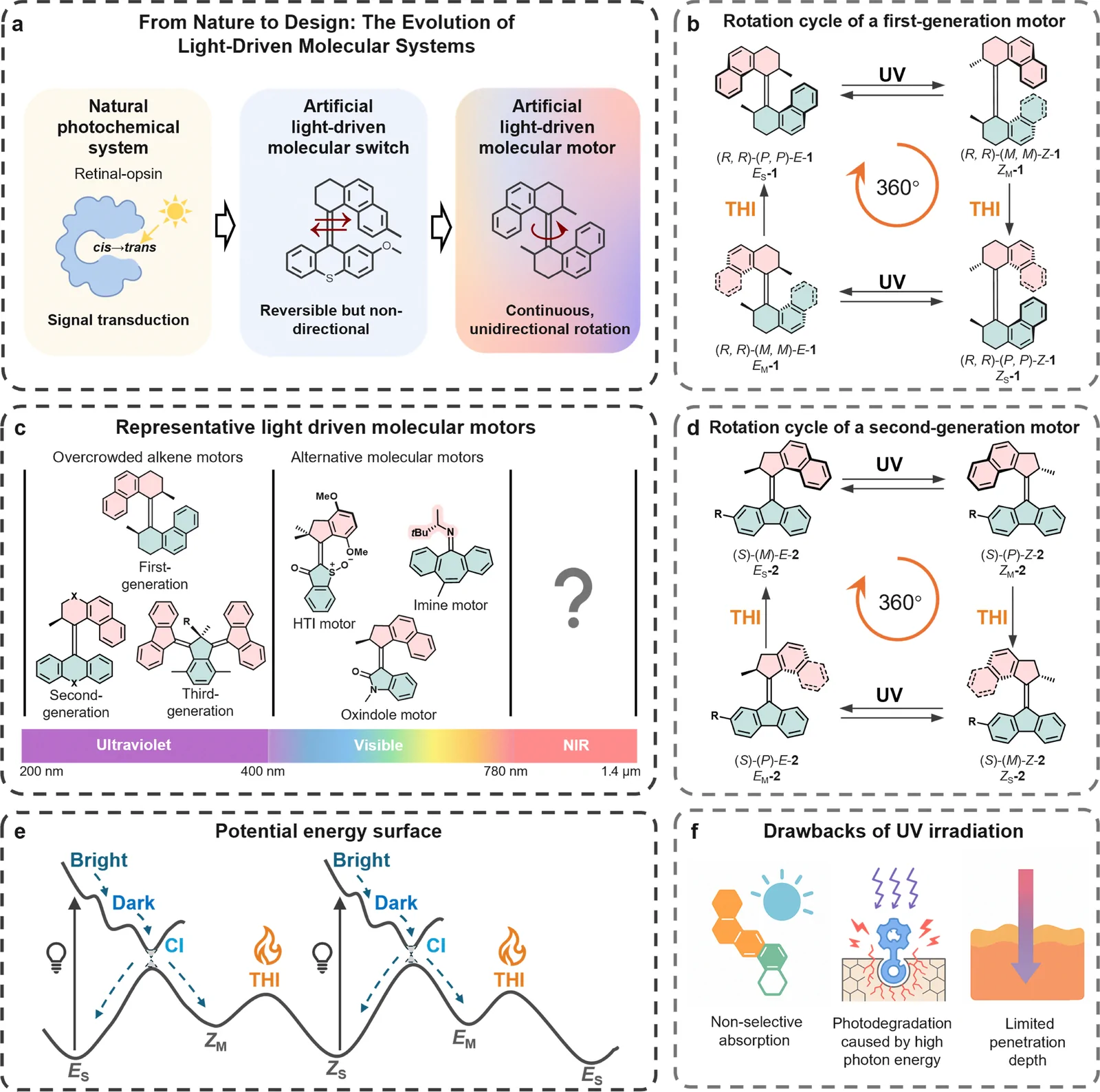
(a) The evolution of light-driven molecular motors, from the natural system through artificial light driven molecular switches. (b) Rotary cycle of a chiral first-generation molecular motor 1. (c) Typical artificial molecular motors. (d) Rotary cycle of a chiral second-generation molecular motor 2. (e) A generic potential energy diagram for light-driven molecular motors. (f) The three major drawbacks using UV irradiation.

The ubiquity and precision of light-driven machinery in nature have long inspired the design of synthetic counterparts that use light to control molecular motion and trigger complex functions. Inspired by these biological systems, the field of synthetic molecular machines has rapidly developed over the past decades, encompassing a wide range of architectures, including mechanically interlocked molecules such as rotaxanes and catenanes, as well as diverse operating principles.^4–10^ Although many types of external stimuli, such as heat, redox processes, and chemical potential, have been employed to drive artificial molecular machines, light remains unparalleled in its spatiotemporal precision. Light allows non-invasive contact-free activation, can be applied locally and instantaneously, and offers tunability of energy input and control through the choice of wavelengths and intensities. Even though photochemical excitation can sometimes lead to side reactions or degradation, it generally provides a clean and non-invasive means of energy input without producing chemical waste. Additionally, photochemical reactions are not constrained by microscopic reversibility, rendering the design of light-driven molecular machines much more straightforward than that of systems driven solely by alternative stimuli.

Building on these advantages of light as an external stimulus, the Feringa group developed photoresponsive overcrowded-alkene-based molecular switches that mimic the reversible photochemical behaviour of retinal, existing as two stable, helically chiral forms that can be interconverted by light (Fig. 1a, middle).^11^ These two states display distinct optical properties, enabling reversible readout by circular dichroism spectroscopy and demonstrating that directionality of switching and change of molecular geometry can be predictably controlled by light. This advance laid the conceptual cornerstone for the development of light-driven molecular rotary motors, in which sequential photochemical isomerisation and thermal helix inversion steps give rise to unidirectional and, in principle, continuous rotation. Since this seminal discovery, numerous generations of photochemical rotary motors featuring diverse molecular scaffolds have been developed, establishing light-driven molecular machinery as a rapidly advancing field. Nevertheless, important fundamental and practical challenges remain, particularly with respect to photostability and the efficient conversion of light-driven molecular motion into useful work. In the context of these challenges, shifting the operational wavelengths of light-driven rotary molecular motors further into visible and near-infrared regions has emerged as a particularly promising strategy, due to the diminished photodamage induced by low-energy light and its compatibility with a broad range of materials and biological tissues. These considerations underscore the timely need for a critical assessment of recent progress in the development of visible-light-driven rotary motors. This tutorial review aims to address the recent developments by highlighting the key design principles, mechanistic insights, and emerging strategies that will shape the next generation of low-energy-light-driven molecular machinery.

### 1.1. Light-driven molecular motors

Among the various light-driven molecular motors developed to date, overcrowded-alkene-based systems have emerged as the archetypical platform. The widespread adoption of this scaffold stems from its straightforward synthesis and modular, tunable structure, which allow the photochemical properties, thermal helix inversion barriers, rotational speed, and function of the motor to be adjusted through structural modification. Equally important is the inherent dynamic chirality and the predictable directional rotary motion of overcrowded-alkene molecular motors, which is encoded in their structure. Steric repulsion between the bulky substituents around the central alkene forces the two halves of the molecule to twist out of the plane of the central C=C bond, giving rise to a helically chiral structure. The preferred handedness of this helical conformation is biased by the point chirality of the adjacent stereogenic center. Thus, the interplay between point and helical chirality gives rise to two conformational states. In the thermodynamically stable isomer, the two stereogenic elements are matched, placing the molecule in a low-energy, often global, minimum on the potential-energy surface. In the metastable isomer, the mismatch between point and helical chirality imposes a more strained, twisted molecular geometry corresponding to a higher-energy local minimum. Along the forward rotational pathway, this metastable state is separated from the subsequent stable isomer by a comparatively low activation barrier associated with thermal helix inversion. By contrast, returning to the preceding stable isomer would require crossing a barrier associated with thermal *E/Z* isomerisation of the central double bond, which is usually substantially higher. Consequently, these motors typically complete a unidirectional full 360° rotation through a four-step cycle consisting of two photochemical isomerisation steps and two thermal helix inversion (THI) steps. Using the first-generation motor developed by the Feringa group as an illustrative example (Fig. 1b and e),^12^ the rotation cycle starts from the thermally stable *E* isomer (*E*_s_). Upon irradiation, photochemical *E* → *Z* isomerisation around the central C=C bond generates the metastable *Z* isomer (*Z*_m_). This metastable state subsequently undergoes thermal helix inversion (THI) to yield the thermally stable *Z* isomer (*Z*_s_). A second photochemical step then induces *Z* → *E* isomerisation, producing the metastable *E* isomer (*E*_m_), which finally relaxes through another THI step to regenerate the stable *E* isomer (*E*_s_). Completion of this four-step sequence corresponds to a full 360° unidirectional rotation.

Subsequent developments led to the second-generation molecular motors in which the two halves of the molecule have distinct structures (Fig. 1c and d).^13–15^ The fragment with the larger moment of inertia is commonly referred to as the stator, whereas the more mobile fragment is termed the rotor, which rotates relative to the stator around the central double bond. This design allows the steric and electronic properties of the rotor and stator fragments to be tuned more independently, enabling improved control over the rotational behaviour. Third-generation motors further modify the stereochemical encoding of directional motion and introduce alternative design strategies that enable more complex rotational behaviour (Fig. 1c).^16^

Beyond the classical overcrowded alkene framework, several other molecular architectures have meanwhile also been developed to achieve light-driven rotary motion. These include, in particular, imine-^17,18^ and hemithioindigo-based motors,^19–24^ developed by the Lehn and Dube groups, respectively, which broaden the structural and mechanistic diversity of artificial molecular motors. The operation and performance of these molecular motors are governed by both photochemical and thermal processes involved in the rotational cycle. In the following sections, these two processes are discussed separately, starting from the photochemical isomerisation step, followed by the thermal helix inversion process.

### 1.2. Photochemical isomerisation steps

Photoisomerisation constitutes the key photochemical step in light-driven molecular motors, which powers the system by converting photon energy into structural change. Upon photoexcitation, isomerisation around the central double bond converts the stable isomer into a metastable state. The efficiency and outcome of this process are commonly described using several experimental parameters.

#### 1.2.1. Absorption and irradiation wavelengths

The interaction between a molecular motor and light is primarily determined by its absorption characteristics. The absorption wavelength, typically expressed as the wavelength of maximal absorption (*λ*_max_), reflects the electronic structure of the chromophore and defines the spectral region where excitation is most efficient. The probability of photon absorption at a given wavelength is quantified by the molar absorption coefficient (*ε*) according to the Beer–Lambert law.

In contrast, the irradiation wavelength (*λ*_irr_) is an externally chosen experimental parameter used to drive the photochemical process. Although excitation near *λ*_max_ often maximises light absorption, the *λ*_irr_ is not necessarily located at the absorption maximum. In many photoisomerisable systems, the absorption bands of the two isomers partially overlap. As a result, irradiation at *λ*_max_ may simultaneously excite both species, reducing the selectivity of the photochemical transformation. Consequently, irradiation wavelengths are often selected in spectral regions where the absorption difference between the isomers is larger, thereby favouring one direction of photochemical isomerisation and improving the resulting photostationary state distribution. This strategy is generally advantageous for photoswitches and for individual photoisomerisation steps of molecular motors. However, during continuous motor operation, complete spectral separation is not always desirable, as substantial spectral overlap between motor intermediates can help maintain efficient photon absorption throughout the entire rotary cycle.

#### 1.2.2. Photoisomerisation efficiency and excited-state dynamics

The efficiency of the photochemical step is commonly quantified by the quantum yields (QYs, *Φ*) of photoisomerisation, defined as the fraction of absorbed photons that lead to photoisomerisation:^25^1
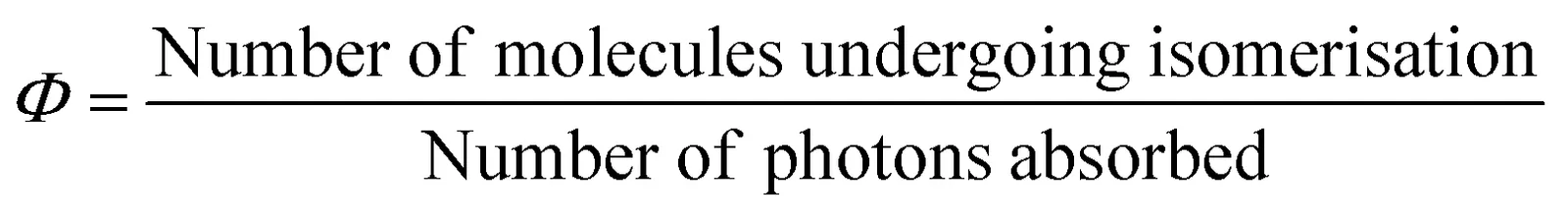


For the Feringa-type overcrowded alkene molecular motors, ultrafast spectroscopic studies have provided a general mechanistic picture of the excited-state processes following photoexcitation (Fig. 1e).^26–28^ Upon light absorption, the molecule is promoted to a Franck–Condon excited state, often described as a bright state. This state rapidly relaxes on the excited-state potential energy surface toward a lower-energy intermediate with a significantly reduced transition dipole moment, commonly described as a dark state, which is often considered a local minimum on the excited-state potential energy surface.

The relaxation from the Franck–Condon region to this intermediate may proceed either barrierless or through a small energetic barrier. Subsequent structural evolution typically involves partial rotation around the central C=C double bond together with pyramidalisation at the adjacent carbon atoms. Motion along these coordinates can guide the system toward regions of strong non-adiabatic coupling between the excited and ground states, frequently associated with conical intersections (CI).^26,27^

Passage through such intersections enables the system to return to the ground-state surface, where relaxation may lead either to the original stable state or to the metastable state. It has been proposed that the accessibility and topology of the conical intersection may influence the quantum yield of photoisomerisation.^29^ The metastable state produced in this process can subsequently undergo a THI step to form the next stable state, thereby completing one half of the rotary cycle.

#### 1.2.3. Photostationary state (PSS)

Under continuous irradiation, photoisomerisation reactions typically reach a dynamic equilibrium known as the photostationary state (PSS). At this stage, the rates of forward and backward photochemical transformations become equal, resulting in a constant distribution of isomers under the given irradiation conditions. This isomer ratio is often referred to as the photostationary state distribution (PSD). The composition of the PSD depends on both the absorption characteristics of the individual isomers and the efficiencies of their respective photoisomerisation processes. Because distinct isomers often absorb light differently at a given wavelength, the irradiation wavelength plays a crucial role in determining the final isomer ratio.

It should be noted that the detailed topology of the excited-state potential energy surface and the precise nature and accessibility of the CIs may depend on the molecular scaffold, substituent pattern, and environmental factors such as solvent polarity. Consequently, although this framework provides a useful conceptual description of the photochemical step in overcrowded alkene motors, the excited-state reaction pathways are not yet fully understood, and their rational control remains an ongoing challenge.

### 1.3. Thermal helix inversion steps

Following the photochemical isomerisation step, overcrowded-alkene-based molecular motors typically undergo a thermal helix inversion (THI), which converts the metastable state into the next thermodynamically stable state and completes half of the rotary cycle. In contrast to the photochemical step, the THI proceeds as a thermally activated process.

The kinetics of this process are commonly characterised by the Gibbs free energy of activation (Δ^‡^*G*), which represents the energy barrier that must be overcome for the metastable isomer to undergo helix inversion.^28^ In overcrowded alkene motors, the magnitude of this barrier is strongly influenced by steric interactions in the so-called *fjord* region around the central double bond. Increasing steric congestion typically raises the inversion barrier and prolongs the lifetime of the metastable state, which is commonly expressed by the half-life (*t*_1/2_), defined as the time required for half of the metastable population to thermally revert to the stable state. Conversely, reduced steric interactions lead to faster thermal relaxation.

In addition to determining the rate of thermal relaxation, the thermal step also plays a central role in establishing the directional bias of the rotary cycle. From the perspective of nonequilibrium thermodynamics, the THI step plays a key role in establishing the directional bias of the motor cycle, as the relaxation from the metastable to the stable state releases free energy that is transduced into mechanical motion. Importantly, this energy dissipation alone does not guarantee directional rotation; rather, in light-driven molecular motors, photochemical excitation removes the constraints of microscopic reversibility, enabling biased flux through the cyclic reaction network, while the direction of rotation is governed by the exergonic ground-state relaxation process. The directional bias of the motor can be quantitatively described by the ratcheting constant *K*_r_, defined as the ratio of the rates of the elementary processes involved in the forward and reverse rotational cycles.^30^ Under simplified conditions, where the total absorbance changes only marginally during the rotary cycle, *K*_r_ can be expressed as:2
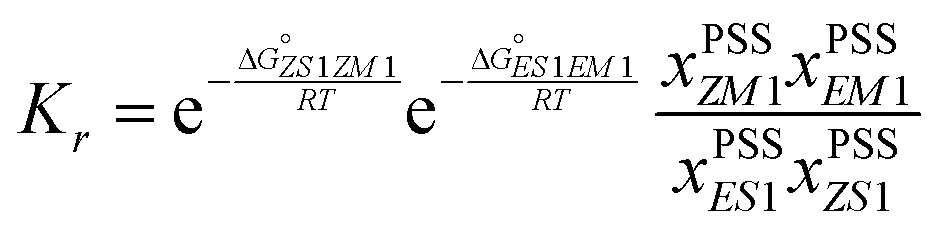
where *x*^PSS^ are molar fractions of the denoted isomers in the respective PSS. Considering that the difference in Gibbs free energy (Δ*G*°) between the corresponding stable and metastable isomers is typically large (≫10 kJ mol^−1^), the *K*_r_ for molecular motors assumes exceedingly high values, far surpassing chemically-driven motors, regardless of the photoconversion to the metastable isomers at both PSSs. Consequently, the unidirectionality of the molecular motor is predominantly biased by the irreversible thermal isomerisation processes. However, detailed kinetic analyses have suggested that the overall rotation rate of molecular motors may not be determined solely by the Δ*G*° between stable and metastable isomers. Instead, it results from the combined influence of thermal activation barriers (Δ*G*^‡^), photochemical quantum yields, molar absorptivities, irradiation conditions, and the coupling between the photochemical and thermal steps under the experimental conditions.^31,32^

### 1.4 Why visible and near-infrared (NIR) light matter?

The photochemical and thermal steps discussed above determine the fundamental operating principles of molecular rotary motors. As mentioned in Section 1.2, the photochemical isomerisation step is initiated by light absorption and therefore depends directly on the excitation wavelength. Extending the absorption and operational wavelength of molecular motors into the visible and NIR regions represents an important objective for improving their practical applicability. The key advantages of longer-wavelength excitation are discussed below.

#### 1.4.1. Improved spectral selectivity and orthogonal activation

One important advantage of extending excitation wavelengths into the visible and NIR regions is the improved spectral selectivity that can be achieved in photoresponsive systems. UV light is absorbed by many common chromophores, which often leads to non-selective excitation in multicomponent systems (Fig. 1f).^26,33^ As a result, competing photochemical processes, energy dissipation, or undesired side reactions may occur, which will be especially critical in more advanced multifunctional molecular machinery.

In contrast, chromophores designed to operate by visible or NIR light can be tuned to absorb within more specific spectral windows. This spectral separation enables orthogonal activation of different photoresponsive units by selecting appropriate wavelengths, allowing multiple light-controlled processes to be addressed independently within the same molecular or materials system. Such wavelength-selective control further provides opportunities for multi-wavelength regulation of complex photoresponsive assemblies.

#### 1.4.2. Reduced photodamage and improved operational stability

Another important advantage of using longer wavelengths is the reduced photodamage associated with visible and NIR light. Compared with ultraviolet irradiation, which carries higher photon energy, visible and NIR excitation generally provide milder operating conditions for photoresponsive systems (Fig. 1f).^34^ This reduced excitation energy lowers the probability of undesired photochemical side reactions, photobleaching, or irreversible degradation of molecular components. As a result, molecular motors driven by visible or NIR light often exhibit improved operational stability, particularly when integrated into soft materials, polymer matrices, or biological environments where prolonged irradiation may otherwise compromise structural integrity.

#### 1.4.3. Improved light penetration and reduced scattering

UV light experiences pronounced Rayleigh-type scattering in condensed media, where the scattered intensity scales with the inverse fourth power of the wavelength (∝ *λ*^−4^). For example, assuming the same density of scatterers, the intensity of Rayleigh scattering of 700 nm light is nearly thirty times lower than that of 300 nm light. As a result, UV light is strongly scattered and absorbed in many environments. In biological tissues, its penetration depth is typically less than 100 µm due to absorption by proteins, nucleic acids, and other biomolecules (Fig. 1f).^35^

In contrast, visible and NIR light penetrate much deeper as scattering and autofluorescence decrease with increasing wavelength.^35^ The so-called “optical or phototherapeutic window” (approximately 650–900 nm, often extended to 1000–1350 nm for the NIR-II region) allows optical transmission through tissues up to several millimetres, around 1–3 mm for NIR-II illumination, enabling high-resolution imaging and photochemical manipulation *in vivo*.^33,36^

Beyond biological systems, this improved penetration is also important for bulk photoresponsive materials. When UV light is used, activation is often limited to the surface region, whereas visible or NIR excitation can enable more efficient activation throughout the material.

#### 1.4.4. Energy utilisation efficiency

Beyond the advantages mentioned above, extending the operational wavelength into the red provides an opportunity to enhance the energy utilisation efficiency of molecular motors. The maximum work that a molecular motor can exert per half-cycle is given by the Gibbs free energy difference between the stable and metastable isomers (Δ*G*°). On a molecular basis, this difference corresponds to Δ*G*°/*N*_A_. Considering the finite quantum yield (*Φ*) of photoisomerisation, which accounts for the fraction of productive photoisomerisation events, the maximum work that can be generated per absorbed photon is governed by *Φ*Δ*G*°/*N*_A_. At the same time, the energy input associated with the absorption of a single photon is determined by the photon wavelength (*hc*/*λ*). Therefore, a simplified expression for the upper bound of this efficiency can be written as:3
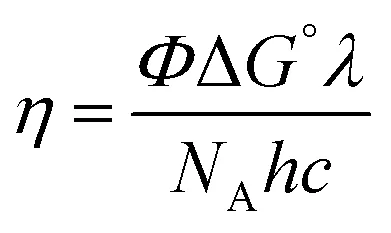
where *λ* is the irradiation wavelength, and *N*_A_ is Avogadro's constant. This expression indicates that the efficiency is governed by both the quantum yield and the ratio between the stored free energy (Δ*G*°) and the photon energy.

Given that Δ*G*° for molecular motors typically lies in the range of 10–40 kJ mol^−1^, the overall efficiency of molecular motors is relatively low, and only a small fraction of the absorbed photon energy is stored in the metastable isomer, while most of the energy is dissipated as heat during photoisomerisation.^26^ Assuming a quantum yield of isomerisation of unity, this efficiency is about 2–10% for excitation with 300 nm light, and rises to 5–23% when using 700 nm light. In practice, however, the actual efficiency is significantly lower due to non-unity quantum yields, competing photochemical pathways, viscous dissipation, solvent reorganisation, and other irreversible losses. These limitations are consistent with general thermodynamic constraints on energy transduction in nonequilibrium molecular systems.

Collectively, these factors make the red shift of operational wavelengths not only desirable but indispensable for achieving controlled and durable photoactivation in complex environments, paving the way for future development of molecular motor systems. Therefore, in this review, we summarize the major molecular design strategies that have been developed to extend photoresponsiveness of molecular motors into the visible and NIR regions. These design strategies can be broadly divided into two categories: direct and indirect approaches. The direct approaches focus on tuning the molecular scaffold to achieve intrinsic, red-shifted absorption, whereas the indirect approaches employ auxiliary sensitizers or chromophores to mediate energy transfer and facilitate long-wavelength excitation. In the final section, we highlight representative applications of visible- and NIR-light-driven molecular motors in soft materials and biological environments, underscoring their growing practical relevance.

## 2. Direct strategies

Direct strategies aim to render molecular motors responsive to visible or NIR light through intrinsic structural modification. The principle is to tune the electronic structure of the motor so that its lowest-energy electronic transition falls within the desired spectral range. This is typically achieved by extending π-conjugation, introducing donor–acceptor substituents to create intramolecular charge-transfer states, or coordinating to metal centers that stabilize low-energy excited states. Through such designs, the absorption can be systematically red-shifted while maintaining the nature of the essential photochemical processes that drive unidirectional rotation.

### 2.1. π-Conjugation extension

Extending π-conjugation is a widely used strategy to red-shift the absorption of molecular motors, as it enhances electronic delocalization across the central alkene and narrows the HOMO–LUMO gap, allowing excitation with lower-energy light.

Representative studies in 2017 and 2019 demonstrated this concept using two π-extended analogues of the second-generation motor 2 (3 and 4, Fig. 2a).^37,38^ In motor 4, incorporation of a pyrene unit into the upper half enlarged the π-system and shifted the *λ*_max_ from 385 nm in the parent motor to 414 nm, enabling operation under 455 nm irradiation. In motor 3, with further extended conjugation by fusing two aryl rings onto the lower half to form a dibenzofluorene motif, an even larger red shift is achieved with a *λ*_max_ of 419 nm and a broad absorption band extending to approximately 550 nm. The PSS ratios remained comparable to those of the parent motor 2.^15^ However, analysis of the photochemical behaviour indicated somewhat reduced photoconversion efficiency. For motor 3, this was inferred from the slower formation of the metastable state, whereas for motor 4 the measured QYs were slightly lower than those of the parent motor, although still within the same order of magnitude.

**Fig. 2:**
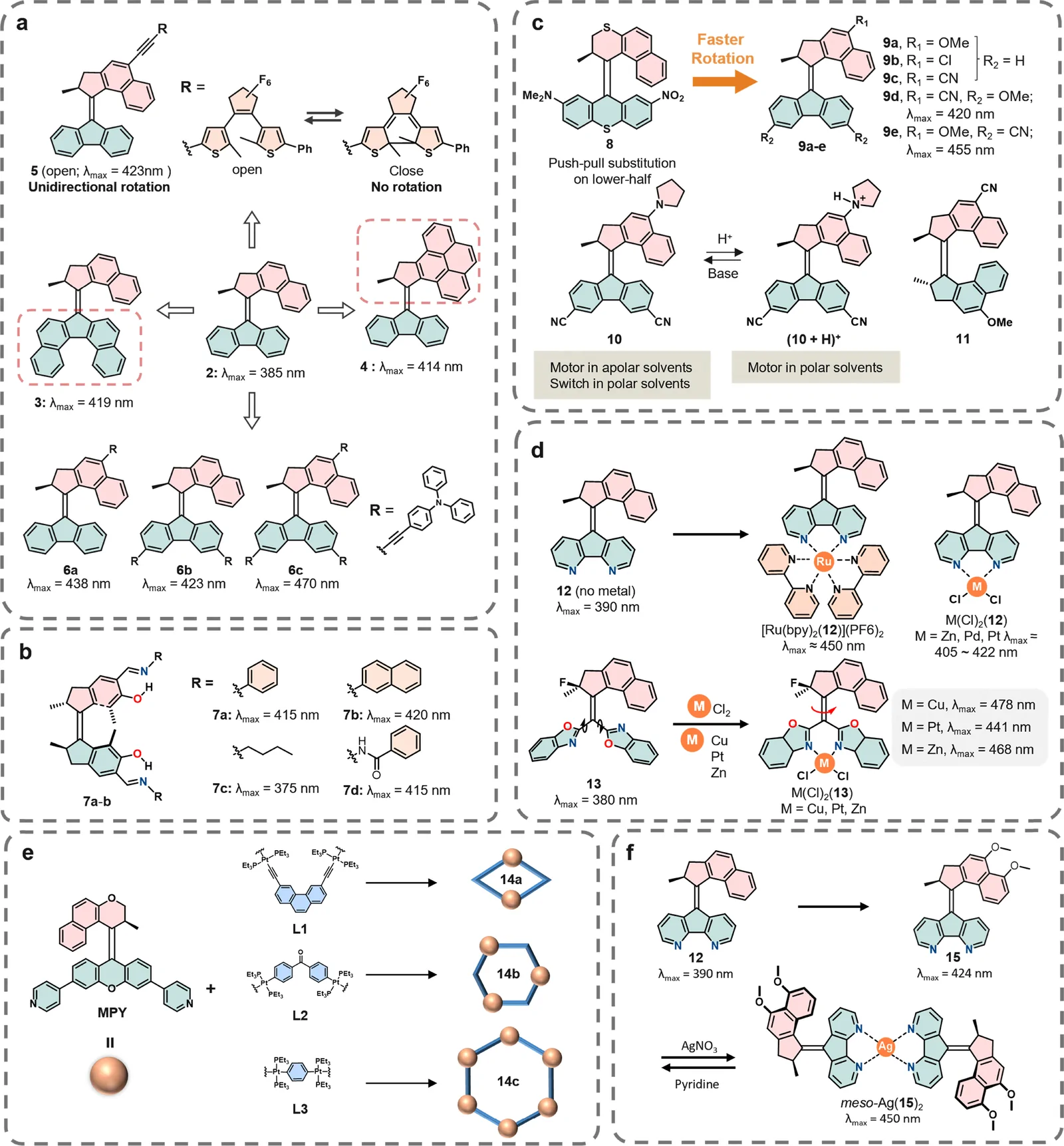
Structural modification of first- and second-generation overcrowded-alkene motors toward visible and NIR light activation upon (a) and (b) Core extension. (c) Push–pull system. (d)–(f) Metal coordination strategies.

Beyond these simple π-extensions, more sophisticated core modifications have been shown to not only red-shift the absorption of molecular motors but also introduce additional optical functionalities. In 2018, the Feringa group reported a dual-function molecular motor, combining second-generation motors and dithienylethene (DTE) photoswitches (5, Fig. 2a).^39^ The DTE unit extends the π-conjugation of the motor, shifting the absorption maximum in CH_2_Cl_2_ from 390 nm in the parent motor to 423 nm, thereby allowing visible-light (455 nm) operation. Additionally, when the DTE is in the open form, the motor performs its normal unidirectional cycle powered with light and heat. Closing the DTE with 312 nm light halts rotation completely, while reopening it with 528 nm light restores the motor function.

Another dual-functional molecular motor was reported by the Feringa group in 2022, where the DTE substituents were replaced with triphenylamine (TPA) groups. These substituents act as strong electron-donating substituents, while simultaneously altering the photophysical properties of the molecular motor core by increasing its two-photon absorption cross-section (6, Fig. 2a).^40^ To systematically examine substitution effects, three motors (6a–c) were synthesized, featuring different TPA arrangements on the upper and/or lower halves of the overcrowded-alkene framework. These modifications progressively extended the π-system, leading to a bathochromic shift of the absorption maximum from 423 nm (6b) to 470 nm (6c) in MeCN.

All three motors undergo efficient formation of the metastable isomer and subsequent thermal relaxation, with PSS ratios of approximately 70 : 30 (metastable :  stable) and activation barriers Δ^‡^*G* between 86–88 kJ mol^−1^. The QYs of the forward photoisomerisation (*Φ*_stable→metastable_) decrease systematically from 15.1% for 6a to 5.2% for 6b and 2.3% for 6c, reflecting that increasing π-conjugation might lower the photoisomerisation efficiency. Importantly, the incorporation of TPA units enables operation not only under visible light (up to 565 nm), but also *via* two-photon absorption (2PA), particularly for motor 6c (*λ*_irr_ = 800 nm) due to its large two-photon absorption cross-section (*σ*_2_ ≈ 1100 GM). In a 2PA process, the molecule simultaneously absorbs two low-energy photons to reach the same excited state accessed by single-photon excitation. This non-linear pathway enables the use of NIR light, allowing excitation with low-energy photons. However, the inherently small two-photon absorption cross-sections of most organic chromophores also imply that high-intensity, tightly focused light sources are typically required, which is incompatible with most biological environments.^33^

Motors 6a–c also exhibit wavelength-dependent emission: 400 nm excitation gives long-lived fluorescence, whereas 445 nm or 800 nm two-photon excitation mainly drives photoisomerisation, suppressing emission. Thus, 6a and 6c allow selective switching between luminescence and rotation, whereas 6b shows both phenomena due to near-degenerate excited states. This dual behaviour may assist in applications requiring separate visualization and actuation.

More recently, this strategy was extended to first-generation motors. In 2024, Schiff-base derivatives were incorporated into both halves of a first-generation overcrowded-alkene scaffold, giving rise to a family of salicylidene Schiff-base functionalized molecular motors (7a–d, Fig. 2b).^41^ These motors combine π-extension with the formation of intramolecular hydrogen bonding between the phenolic hydroxyl group and the imine nitrogen, which is associated with pronounced red shifts in the absorption spectra. As a consequence, the *λ*_max_ are markedly red-shifted compared with the parent motor, reaching approximately 415 nm for 7a and 7d, 420 nm for 7b, and 375 nm for 7c, depending on the aryl substituent introduced at the Schiff-base position. The more conjugated naphthyl-substituted derivative 7b exhibits the largest red shift and the highest PSS selectivity under 420–470 nm irradiation.

In addition to their red-shifted absorption, motors 7a–d exhibit pronounced impact of solvent and temperature on their photoisomerisation. Polar protic solvents stabilize the OH tautomer and lead to higher PSS values and more efficient switching, whereas non-polar media favor the NH-form and significantly reduce the switching efficiency. Similarly, lower temperatures enhance photoisomerisation, while higher temperatures shift the OH–NH equilibrium toward the NH tautomer and decrease the PSS.

### 2.2. Push–pull systems

Push–pull electronic systems are widely employed in various photoactive molecules such as molecular switches and fluorescent dyes.^42,43^ In these cases, electron-donating (D) and electron-withdrawing (A) groups positioned across a π-conjugated framework induce intramolecular charge-transfer (ICT) transitions, effectively narrowing the HOMO–LUMO gap and shifting both absorption and emission toward longer wavelengths.

The first visible-light-driven molecular motor based on a push–pull design was reported in 2003 (8, Fig. 2c).^44^ In this work, a strong electron-donating group (–NMe_2_) and an electron-withdrawing group (–NO_2_) were introduced into the lower half of a second-generation motor. This substitution substantially reduced the HOMO–LUMO gap, shifting the absorption maximum into the visible region at around 435 nm. The same modification was also associated with an increased rate of thermal isomerisation. While the red-shifted absorption primarily arises from electronic effects within the donor–acceptor framework, the acceleration of thermal isomerisation likely involves additional steric and conformational factors in the motor scaffold. Furthermore, protonation experiments revealed that the motor's photochemical behaviour could be modulated by acid stimuli, highlighting the potential of combining electronic push–pull effects with external regulation (Fig. 2c). This study established donor–acceptor engineering as a powerful strategy to achieve red-shifted activation and laid the foundation for subsequent developments in visible-light-responsive molecular motors, as discussed below.

Although this study convincingly demonstrated that donor–acceptor substitution is an effective strategy for visible-light-driven rotation, early second-generation motors still exhibited relatively high thermal barriers for the THI steps, resulting in slower rotation speed. To improve the rotational dynamics, the Feringa group redesigned the core scaffold by replacing the six-membered ring with a five-membered ring in the upper half, while converting the lower half into a fluorene unit.^13,15^ This modification reduced steric congestion around the central alkene and altered the electronic structure of the motor scaffold, thereby lowering the activation barrier of the THI steps and accelerating the rotary motion.

Building upon this improved scaffold, later studies systematically explored how fine-tuning the electronic environment around the central double bond affects both photochemical and thermal steps. In particular, a 2008 study examined the motors with electron-donating (–OMe) and electron-withdrawing (–Cl, –CN) substituents appended to the upper half in a position directly conjugated to the C=C axis (9a–c, Fig. 2c).^45^ While both types of substituents induced a modest red-shift of the π–π* absorption band (*λ*_max_ ≈ 410–430 nm), their influence on the kinetics of thermal process were opposite: donor groups (9a) slightly reduced the activation barrier (Δ^‡^*G* ≈ 92 kJ mol^−1^) and accelerated the THI rate, whereas acceptor groups (9b and c) increased the barrier (Δ^‡^*G* ≈ 95–97 kJ mol^−1^) and prolonged the stability of the metastable isomer (*t*_1/2_ ≈ 3–4 h). The photoisomerisation QYs (*Φ*_s→m_ ≈ 5–6%) and photostationary ratios (PSS ≈ 80–85% of metastable isomer) remained largely unchanged.

In 2019, they further explored this strategy by installing substituents in both upper and lower halves, in conjugation with the central double bond serving as the axle of the motor (9d and e).^46^ In this design, the upper and lower halves bear *p*-methoxy (–OMe) and cyano (–CN) groups in conjugation with the central C=C bond, generating a well-defined donor–acceptor polarity across the molecular axle. TD-DFT calculations and UV-vis spectroscopy indicate that the donor–acceptor interaction narrows the HOMO–LUMO gap, red-shifting the *λ*_max_ from 422 nm for 9d to 453 nm for 9e. Notably, 9e remains photoresponsive even under green-light irradiation up to 530 nm.

Besides this photophysical improvement, the push–pull substitution also influences the kinetics of the thermal steps. Motor 9e exhibits a higher activation barrier for the thermal helix inversion (Δ^‡^*G* ≈ 91 kJ mol^−1^) compared with 9d (Δ^‡^*G* ≈ 86 kJ mol^−1^), resulting in slower thermal relaxation. DFT calculations indicate that the structural differences responsible for this variation originate primarily from the transition state of the THI process, whereas the geometries of the metastable isomers are nearly identical. Consequently, 9d undergoes faster thermal rotation but with slightly reduced light responsiveness. Both motors maintain comparable photoisomerisation QYs (*Φ*_s→m_≈ 5–12%) and PSS compositions of approximately 75–80% of the metastable isomer.

In 2022, Pfeifer *et al.* synthesized a push–pull substituted second-generation molecular motor whose operation mode can be reversibly controlled by both protonation and solvent polarity (10, Fig. 2c).^47^ In motor 10, a pyrrolidino donor on the upper half and two cyano acceptor moieties on the lower half establish a strong donor–acceptor conjugation across the central C=C bond, resulting in strong absorption across the spectrum and efficient photoisomerisation under green light irradiation up to 528 nm. In addition to these photophysical improvements, the push–pull electronic structure also renders the motor highly responsive to its environment. In nonpolar solvents, the unprotonated molecule exhibits unidirectional rotary motion, whereas in polar media, stabilization of the charge-separated state lowers the barrier for thermal isomerisation, transforming the system into a bidirectional “switch mode.” Upon protonation ((10 + H)^+^), the donor strength of the pyrrolidino group is suppressed, effectively cancelling the push–pull character and restoring unidirectional rotation even in polar solvents.

Compared with second-generation motors, the use of the push–pull strategy in first-generation molecular motors was investigated at a later stage and remains less explored, primarily because the upper and lower halves of the first-generation core are identical, making the selective installation of differentiated donor–acceptor groups synthetically more challenging.

Nevertheless, Roy *et al*. overcame this hurdle by constructing a first-generation overcrowded-alkene-based motor bearing a methoxy donor and cyano acceptor directly conjugated to the central C=C axle (11, Fig. 2c).^27^ The *λ*_max_ of 11 shifts drastically from 365 nm in the parent motor to 402 nm in cyclohexane and 415 nm in methanol. This donor–acceptor design places the primary π*–*π* transition in the visible region and enables efficient green light excitation. Besides the red-shifting, this donor–acceptor design gives the excited state a stronger charge-transfer character, which changes the shape of the excited state potential energy surface and its intersection with the ground state potential energy surface along the reaction coordinate. As a result, the QYs of the *Z → E* photoisomerisation depend strongly on the solvent—it is almost 100% in non-polar cyclohexane but drops to about 5% in polar methanol. Transient absorption spectroscopy showed that 11 reaches this conical intersection in less than 50 fs without forming a separate dark state, explaining its high efficiency in non-polar solvents and the strong suppression in polar ones.

Compared with π-conjugation extension, the push–pull strategy offers a more convenient and less intrusive strategy for red-shifting absorption. The substituents required for donor–acceptor modulation introduce minimal steric and conformational perturbations to the motor scaffold, preserving the key photochemical and thermal characteristics of the parent system. More importantly, motors such as 8 and 10 exemplify how donor–acceptor motifs can couple rotation with redox or other orthogonal functions to light triggers, underscoring the promise of this strategy for multiresponsive, functionally integrated molecular systems.

### 2.3. Metal coordination

Covalent modification can tune photochemical properties, but non-covalent interactions provide an alternative with the benefit that the effects are readily tuneable and can be induced reversibly. Among these interactions, metal coordination is an attractive strategy because ligand–metal binding can adjust electronic coupling and excited-state behaviour. When a photoactive ligand coordinates to a transition-metal center, new charge-transfer pathways appear, such as metal-to-ligand charge-transfer (MLCT) and ligand-to-metal charge-transfer (LMCT). These processes often shift the absorption toward the visible region. Over the past decade, transition metals including Ru^2+^, Zn^2+^, Pd^2+^, Pt^2+^, and Cu^+^ have been combined with ligands such as bipyridine, phenanthroline, porphyrin, and diazafluorene to build a variety of photoresponsive systems.

The earliest example was reported by Wezenberg and Feringa in 2015, when they redesigned a second-generation motor by replacing its lower half with a 4,5-diazafluorenyl unit capable of coordinating to metal ions (12, Fig. 2d).^48^ The resulting motor ligand was complexed with a Ru(bpy)_2_ center to form a well-defined Ru(ii) complex. This complex showed strong MLCT absorption in the visible region, with clear bands around 425 and 450 nm, which allowed the motor to be driven by blue light at 450 nm. Metal coordination also changed the geometry of the lower half. Binding to Ru^2+^ caused the diazafluorenyl unit to contract, which reduced steric hindrance around the alkene axle. This effect lowered the thermal helix inversion barrier by *ca.* 10 kJ mol^−1^ and increased the rotation rate roughly fifty times.

It was further demonstrated that metal coordination can also regulate the rotational speed of molecular motors by coordinating motor 12 with different transition metals—Zn^2+^, Pd^2+^, and Pt^2+^.^49^ Metal binding reduced the steric congestion between the two halves of the motor, and this structural change lowered the thermal helix inversion barrier from 81.5 kJ mol^−1^ for the Zn complex to 75.1 kJ mol^−1^ for the Pt analogue. Among these complexes, Pt(Cl)_2_(12) induced the largest bathochromic shift of the π*–*π* absorption band, with *λ*_max_ moving from 405 nm for the Zn and Pd complexes to 422 nm for the Pt complex. Although the red-shift was less pronounced than that observed for the Ru(ii) system, the complexes could be formed *in situ* and remained fully reversible, since the metal ions could be removed by adding a stronger ligand such as pyridine.

Challenging the conventional view that metal coordination merely tunes absorption or rotation rate, Stindt *et al.* demonstrated that complexation can even activate or deactivate the rotary function itself. Their bis(benzoxazole)-based motor, which was too flexible to sustain a simple well-defined directional rotation cycle in its free form (*λ*_max_ = 388 nm), became a fully functional conventional visible-light-driven motor upon coordination with transition metals— Zn^2+^, Cu^2+^, or Pt^2+^ (13, Fig. 2d).^50,51^ Complexation rigidified the lower half and simultaneously induced a pronounced bathochromic shift, with *λ*_max_ at 468 nm (Zn), 478 nm (Cu), and 441 nm (Pt).

More complex architectures have recently been developed to organize multiple motor units through metal-assisted self-assembly. Shi, Qu, Yang and Feringa constructed a family of supramolecular metallacycles (14a–c, Fig. 2e) in which pyridyl-functionalized molecular motors were linked by di-Pt(ii) acceptors into discrete coordination frameworks.^52^ Formation of these Pt(ii)-based assemblies resulted in a red-shift of the motor's π*–*π* absorption band from 370 to 410 nm. Control experiments showed that this shift originates from changes within the electronic structure of the motor ligand rather than from metal-centered charge-transfer transitions. Although the red-shift is relatively small, it enables the metallacycles to be activated by 420 nm light. Importantly, integration into the coordination framework did not interfere with the intrinsic rotary cycle. Spectroscopic and kinetic analyses confirmed that both the photochemical isomerisation and the THI proceeded with efficiencies similar to those of the free motor. In this system, the Pt(ii) centers mainly function as structural connectors that spatially organize several motor units into a defined supramolecular architecture while preserving their fundamental photochemical behaviour.

Li *et al.* designed a coordination-directed self-assembly based on the modified motor ligand 15, in which two methoxy groups were introduced on the upper half of the parent motor 12 to enhance its charge-transfer character and shift its maximum absorption to 420 nm (Fig. 2f).^53^ Upon coordination with Ag(i) ions, two molecules of 15 were linked through a single Ag center to form a well-defined supramolecular dimer, *meso*-Ag(15)_2_. The resulting complex exhibited broad intra- and inter-ligand charge-transfer absorption bands in the 450–500 nm region and showed efficient, reversible visible-light-driven rotation. Although the Ag–N coordination provided sufficient stability for the dimer to exist as a discrete species, the assembly could be cleanly dismantled when a stronger ligand such as pyridine displaced Ag(i), demonstrating that the structure is reversibly maintained through competitive coordination.

Overall, metal coordination provides a modular and reversible means to adjust the photochemical behaviour and rotational dynamics of molecular motors. Depending on the metal–ligand combination, coordination can introduce visible-light responsiveness, alter thermal barriers, or even activate otherwise non-functional scaffolds to function as light-driven motors. Although the extent of tuning varies across systems and is not universally predictable, the structural flexibility and orthogonality of coordination chemistry renders it a valuable complementary method to covalent modification in expanding the design space of light-driven molecular motors.

### 2.4. New designs

Beyond substituent modification, a radically different approach is altering the molecular framework itself by incorporating new chromophoric elements into the motor core, thus achieving visible-light activity as an intrinsic property of the scaffold. Incorporating motifs such as thioindigo, oxindole/isoindigo, barbiturate-type acceptors or formyl groups creates hybrid chromophores whose intrinsic donor–acceptor polarity and extended π-systems enable efficient visible-light activation. These redesigned scaffolds typically absorb in the 400–520 nm region while preserving directional rotation, offering a tunable balance between photochemical efficiency and metastable state stability.

#### 2.4.1 HTI-based motors

Hemithioindigo (HTI) based molecular motors evolved from the excellent visible-light responsiveness of HTI chromophores, which have long been recognized as efficient visible-light-driven photoswitches. The Dube group demonstrated that incorporating the HTI chromophore into an overcrowded alkene framework enables the construction of molecular motors that can be directly activated by visible light (Fig. 3a).^19^

**Fig. 3:**
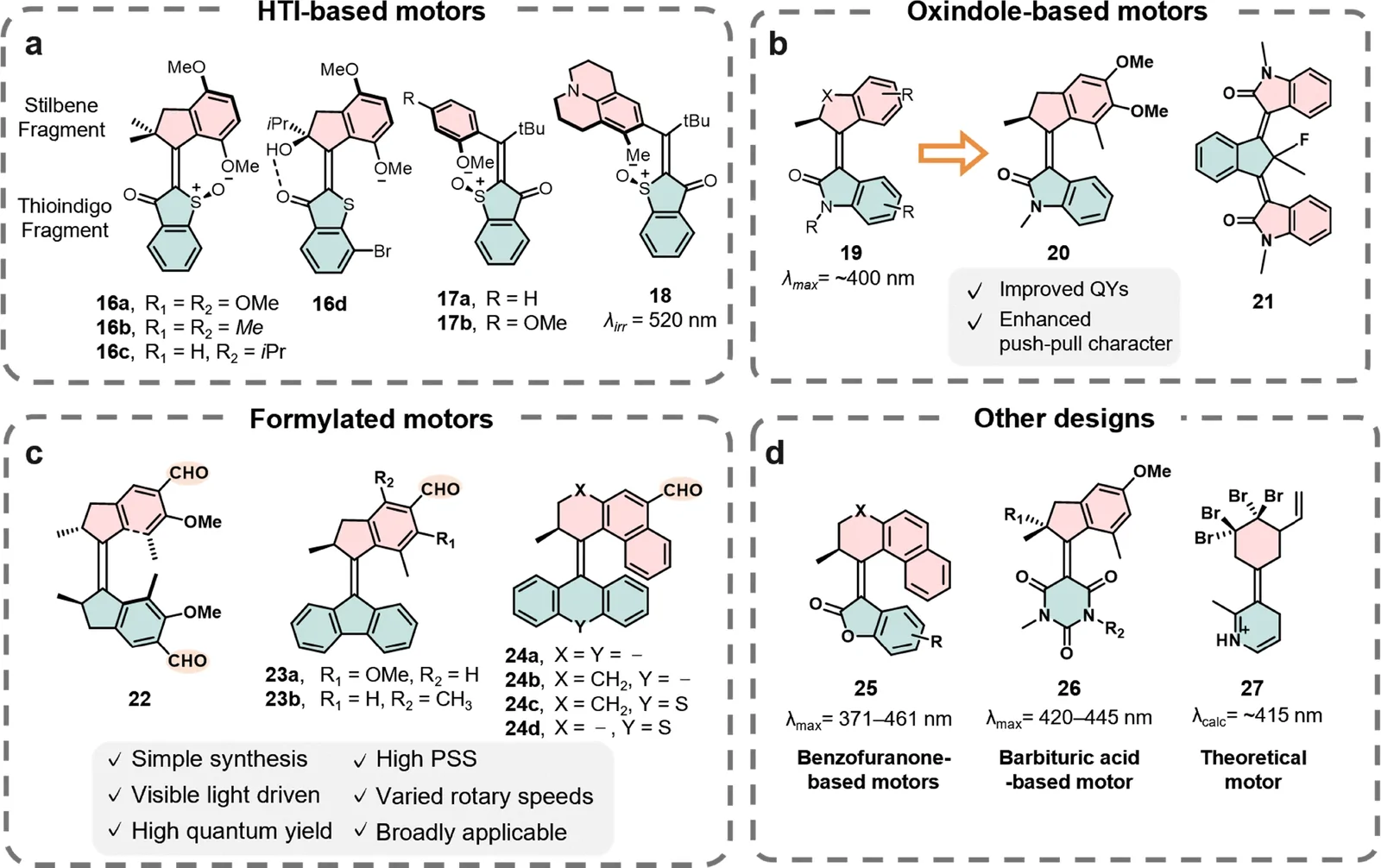
(a) Structures of hemithioindigo (HTI)-based molecular motors. (b) Structures of oxindole-based molecular motors. (c) Structures of formylated molecular motors. (d) Other designs, including benzofurane-based motors, a barbituric acid-based motor and a theoretical motor.

This hybrid architecture combines the central C=C rotary axis of classical Feringa motors with the inherent donor–acceptor character of the hemithioindigo chromophore, resulting in motors that respond to visible light in the 405–505 nm region (16a–c, Fig. 3a).^19,20^ The HTI unit contributes both a polarized chromophore and a stereogenic sulfur center, together generating a helically chiral C=C axis and four distinct isomers. These states interconvert through a sequence of photoisomerisation and thermal helix inversion steps, completing a full 360° rotation cycle. Under visible-light irradiation, motor 16a rotates unidirectionally in the clockwise direction and can reach rotational frequencies up to 1 kHz at room temperature.

Subsequently, the Dube group developed a series of “photon-only” HTI motors in which directional motion is achieved exclusively through photochemical steps, without relying on the thermally activated steps characteristic of conventional Feringa-type motors (17a and b, Fig. 3a).^21,22^ In this system, the geometry evolves in a cycle of photoreactions following a sequence of single-bond rotation (SBR), hula-twist (HT), and double-bond isomerisation, giving rise to unidirectional rotation driven solely by 405 or 442 nm visible-light irradiation.

They also explored how molecular geometry could be used to encode new types of motion. This effort culminated in a green-light-driven motor that follows a figure-eight trajectory, representing a conceptual shift towards more complex spatially controlled motion (18, Fig. 3a).^23^ The molecule incorporates both a sulfoxide stereocenter and an atropisomeric julolidine axis, enabling four isolable diastereomeric states. Its operation proceeds through a four-step sequence alternating photoinduced double-bond isomerisation (DBI) and thermal hula-twist (HT) isomerisations, each contributing to a fixed, unidirectional pathway driven by visible light (*λ*_irr_ = 520 nm). The system also features fluorescence readout, with emissive and non-emissive states corresponding to specific intermediates, offering prospects for photonic information devices and next-generation motor architectures.

More recently, Dube and co-workers reported a new HTI motor in which the source of asymmetry was shifted from the sulfoxide stereogenic center used in earlier HTI motors to a carbon-based stereocenter located on the indanone rotor, while a neighbouring alcohol group enables intramolecular hydrogen bonding with the stator (16d, Fig. 3a).^54^ The sulfur atom in the thioindigo fragment remains unoxidized, a structural feature that the authors expected to produce a notable bathochromic shift relative to established HTI motors.^19^ The motor operates through an unusual five-step cycle in which light-induced double-bond isomerisation is coupled to constitutional alteration, intramolecular epoxide formation and reversible proton transfer, generating a high-energy intermediate.

#### 2.4.2 Oxindole motors

Inspired by Dube's hemithioindigo-based design, the Feringa group has reported a series of oxindole-based molecular motors since 2019 (19 and 20, Fig. 3b).^55–57^ These motors exhibited red-shifted absorption bands (*λ*_max_ ≈ 400 nm) and were capable of being driven at wavelengths up to 505 nm (19). However, despite their visible-light operation, the initial oxindole motors exhibited relatively low photoisomerisation QYs (*Φ* ≈ 2–3%) due to the weak CT (charge-transfer) nature of the excited S_1_ state. Motivated by natural photosystems such as rhodopsin, where high QYs (67%) stem from CT-dominated excited states, the Feringa group sought to strengthen the push–pull polarization within the motor framework.

In 2021, they redesigned the molecule by replacing the rigid fluorenyl-type rotor with a dimethoxybiphenyl upper half while retaining the oxindole stator (20).^56^ This modification established a donor–acceptor-polarized π-system with enhanced CT character in the S_1_ state. As a result, the motor exhibited a pronounced bathochromic shift (*λ*_max_ = 510 nm) and a fourfold enhancement in QYs (*Φ* ≈ 8–12%). Time-resolved spectroscopy and DFT analysis confirmed that enhanced CT coupling facilitated faster photoisomerisation and lowered the thermal helix-inversion barrier, thus achieving a better balance between efficiency and driving wavelength.

Recently, a bridged-isoindigo framework was introduced that extends π-conjugation across two oxindole rotor halves into a third-generation molecular motor (21, Fig. 3b).^58^ This heteroatom-rich design replaces the classical fluorene rotors of third-generation motors, producing an intrinsically red-shifted absorption band (*λ*_max_ = 455 nm) and enabling operation under visible-light irradiation up to 530 nm. Beyond enhancing solubility and band separation, the system exhibits an intrinsic coupled motion, which adds a new dimension to the motor operation, as the rotation of one of the rotors influences the other, enabled by a mutual interaction between the two units and mediated by the formation of a unique double metastable state that bridges their rotational cycles.^58^

#### 2.4.3 Formylated motors

Besides extensive chromophoric redesign, a complementary approach enables substantial enhancement through minimal synthetic alteration. The incorporation of a single formyl group (–CHO) into the π-system effectively modulates the excited-state landscape while preserving the original motor framework.

In 2024, the Feringa group first demonstrated how formyl substitution influences both first- and second-generation overcrowded-alkene-based motors (22 and 24a, Fig. 3c).^59^ Introducing a carbonyl group directly conjugated to the central C=C axle produces a pronounced red shift (≈40 nm) and, more importantly, fundamentally improves the photochemical step of the rotary cycle. The formyl unit acts as a strongly localized π* acceptor that narrows the HOMO–LUMO gap and enhances absorption, but leaves the THI barriers essentially unchanged compared to the parent motor. The formylated motors exhibit a dramatic increase in the forward photoisomerisation quantum yield (up to 80%) accompanied by a 20-fold suppression of the competing backward reaction (≈4%), enabling near-quantitative PSS compositions even under relatively low photon flux.

Although the exact origin of the performance boost remains unclear at present, the authors showed that it cannot be attributed to a simple electronic substituent effect. Control motors bearing other electron-withdrawing groups failed to reproduce the same behaviour, indicating that formylation influences the photochemical step through a more complex combination of structural and excited-state factors. Thus, this study established formylation as a powerful strategy that selectively strengthens the photochemical step without perturbing thermal barriers, marking an important fundamental step for future wavelength red-shifting and performance optimization of overcrowded-alkene-based motors by engineering the excited-state energy landscape. A subsequent study further demonstrated that *para*-ketone and *para*-cyano substituents can also retain excellent photoefficiency, while *meta*-substitution provides an effective handle for tuning the rotary speed without compromising the photochemical performance.^60^ Importantly, these highly photoefficient motors also exhibit efficient sunlight-driven operation, suggesting that enhancing photoselectivity, rather than simply extending the absorption wavelength, represents an effective strategy for developing visible-light-responsive molecular motors.

Building on these achievements, the approach was expanded in 2025 into a general formylation strategy applicable to a variety of overcrowded alkene scaffolds (23 and 24, Fig. 3c).^61^ Motors featuring fluorene-, indanone-, thioxanthene-, and dihydronaphthalene-based lower halves were uniformly improved in a simple two-step sequence of lithium–halogen exchange followed by a DMF quench, confirming the broad compatibility of the modification. Across these frameworks, formyl substitution induced red shifts of 20–40 nm, extending the absorption onset into the visible range (*λ* ≈ 405–420 nm). Moreover, the QYs and conversion to the metastable isomers were consistently increased two- to three-fold, while the ground-state rotational barrier and four-step unidirectional cycle remained unchanged.

Collectively, these results demonstrate that formylation serves as a general and effective strategy to enhance photochemical performance while preserving the intrinsic rotary mechanism across diverse motor structures.

#### 2.4.4 Other designs

In 2022, Zhang *et al.* developed a series of benzofuranone-based motors (25, Fig. 3d).^62^ The oxygen substitution increased the electron-withdrawing character of the lower half and π-delocalization across the furanone ring, leading to red-shifted absorption (*λ*_max_ = 440–470 nm) and unrestricted rotation in polymeric films. Depending on the donor strength of the upper half, motors exhibited tunable CT intensity, with strongly polarized systems (bearing *N*,*N*-dimethylamino donors) achieving rotation under green-light excitation (*λ*_irr_ = 550 nm).

Meanwhile, Feringa and Crespi introduced a barbituric-acid-based stator (26, Fig. 3d).^63^ The strongly electron-withdrawing nature of this unit amplified push–pull polarization, shifting absorption to the visible region (*λ*_max_ = 420–445 nm). During the synthesis, an unexpected α-hydroxylation generated a tertiary stereocenter that formed an intramolecular hydrogen bond with one carbonyl, establishing a hydrogen-bond-assisted “lasso” mechanism for ultrafast *E*/*Z* isomerisation. Femtosecond spectroscopy revealed sub-picosecond switching with quantum yields up to 68%.

Beyond structural and mechanistic innovations, Wang and Durbeej provided a purely theoretical perspective on the fundamental requirements for visible-light-driven molecular rotation (27, Fig. 3d).^64^ Through high-level CASSCF/CASPT2 and non-adiabatic dynamics simulations, they demonstrated that even a minimal Schiff-base framework can undergo complete unidirectional rotation solely under visible-light excitation (*λ*_irr_ ≈ 410–450 nm) without any thermal steps. The model exhibits sub-picosecond photoisomerisation and achieves a fully photochemical two-step rotary cycle. This work does not propose a new scaffold but rather establishes a conceptual boundary, showing how minimal electronic design can yield fast and efficient visible-light-driven molecular motors. Positioned at the theoretical limit of motor design, it provides an insightful complement to experimental advances and a foundation for future computationally guided development.

In the above section, we outlined a range of framework-level design strategies that move beyond simple substituent effects and fundamentally reconfigure how visible-light responsiveness is encoded in molecular motors. HTI-, oxindole-, isoindigo- and benzofuranone-based scaffolds demonstrate the way heteroatom-rich chromophores and strengthened push–pull polarization can red-shift absorption and unlock new photochemical pathways, including fully photochemical cycles and multidimensional trajectories. In contrast, formylated motors exemplify a complementary minimalist strategy, in which a simple modification can markedly enhance photoisomerisation efficiency without perturbing thermal barriers. This approach highlights how subtle electronic modulation, rather than extensive structural redesign, can effectively improve motor performance. Collectively, these diverse architectures illustrate that visible-light-driven rotation can emerge from multiple, parallel design principles, greatly expanding the structural and mechanistic landscape available for next-generation molecular motors.

## 3. Indirect strategies

Natural light-harvesting systems provide clear precedents for energy-transfer sensitization, in which an appended chromophore captures light and relays its excitation to a reactive center. A well-studied example is xanthorhodopsin, a microbial retinal protein that contains a carotenoid antenna (*e.g.*, salinixanthin) capable of absorbing visible light and transferring the excitation to the retinal chromophore, thereby triggering its photoisomerisation with remarkable efficiency (up to 40% energy-transfer yield).^65^ Another classic case is the photosynthetic antenna complex in plants and algae, where pigments such as chlorophylls and carotenoids harvest a broad spectrum of sunlight and funnel the excitation energy to the chlorophyll reaction centers for charge-separation chemistry.^66^ These natural paradigms illustrate how chromophores can broaden the excitation range and efficiently channel energy, inspiring indirect strategies for visible-light activation not only of molecular motors but also of molecular switches, rotaxanes, and other supramolecular systems.

### 3.1. Energy transfer from chromophores

Energy transfer itself can occur through several different mechanisms, the most classical being Förster resonance energy transfer (FRET) and Dexter exchange. The former operates through long-range dipole–dipole coupling between singlet excited states and is efficient over distances of 10–100 Å, provided there is sufficient spectral overlap between the donor's emission and the acceptor's absorption.^67^ In contrast, Dexter-type transfer is a short-range electron-exchange process that requires orbital overlap between the donor and the acceptor and is therefore effective only within ∼10 Å.^68^ These two modes represent the limiting cases of long-range and short-range energy transfer mechanisms, while most experimental systems fall somewhere between them.

Cnossen *et al.* and Pfeifer *et al.* implemented these two mechanisms in the design of molecular motors driven by visible light. The former synthesised the first green-light-driven overcrowded-alkene-derived motor by connecting a palladium tetraphenylporphyrin (PdTPP) sensitizer to a second-generation motor. This design enabled efficient triplet–triplet energy transfer (TTET) from the triplet state of PdTPP to the motor core, thereby replacing ultraviolet excitation with green light (*λ*_irr_ = 532 nm) (28, Fig. 4a).^69^ The latter extended the excitation wavelength to the NIR regime by attaching a TPA chromophore (AF-343) through a flexible, non-conjugated linker, where excitation energy was transferred *via* Förster-type coupling from the dye to the motor's singlet excited state (29, Fig. 4a).^70^

**Fig. 4:**
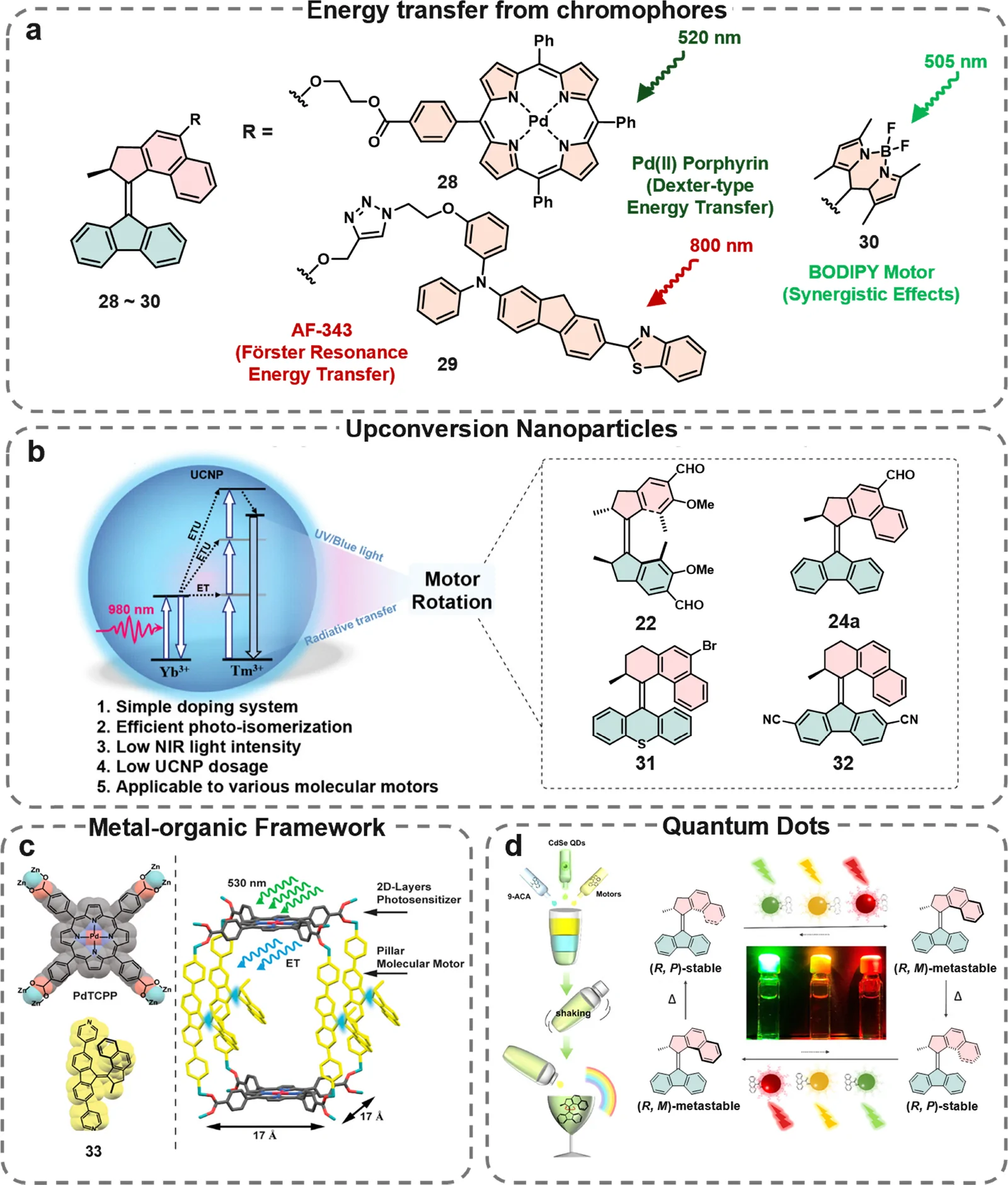
(a) Structures of visible- or NIR-light-driven molecular motors *via* covalent attachment to chromophores (28–30). (b) General strategy to power rotary molecular motors and switches by NIR light through UCNPs. Adapted with permission from ref. 72. Copyright 2025, American Chemical Society. (c) Structures of PdTCPP and bispyridyl molecular motor 33 used as linkers for construction of the MOF framework, and schematic representation of the rotation of molecular motor 33 incorporated as struts in the motorized pillared-paddlewheel PdTCPP MOF driven by energy transfer from a PdTCPP sensitizer (right). Relevant approximate dimensions of the unit cell are given. Adapted with permission from ref. 74. Copyright 2020, American Chemical Society. (d) Powering molecular motors under the rainbow using quantum dots (QDs). Adapted with permission from ref. 75. Copyright 2025, American Chemical Society.

Together, these studies illustrate how complementary Dexter and Förster pathways can be harnessed to achieve long-wavelength photoactivation of molecular motors. More importantly, both studies underscore the intrinsic limitations of the respective underlying mechanisms. In the case of TTET, the extent of the red shift in the excitation wavelength is constrained by the energy of the triplet excited state of the molecular motor. While this energy varies strongly depending on the structure, it is roughly estimated at 180 kJ mol^−1^ for the archetypal second-generation molecular motor core 2. Considering that efficient TTET requires favourable thermodynamics, the maximum excitation wavelength for the molecular motor achievable under this mechanism is limited to approximately 665 nm. Conversely, while the resonant energy transfer imposes virtually no constraints on the excitation wavelength, this approach suffers predominantly from the low efficiency of the photochemical excitation of the motor core, particularly when 2PA is employed.

On the other hand, Toyoda *et al.* integrated a bright photoluminescent dye directly into the motor architecture, yielding a BODIPY–motor hybrid that unites rotary motion and emission within a single molecule (30, Fig. 4a).^71^ In contrast to the previous sensitised systems, the BODIPY unit was directly attached to the upper half of a second-generation motor through a single C–C bond, enforcing an almost perpendicular spatial arrangement between the two moieties. This configuration allowed moderate π*–*π interaction while preserving both photoluminescent and rotary functions of the hybrid. Upon 395 nm irradiation, the hybrid displayed normal *E → Z* photoisomerisation and thermal helix inversion; remarkably, identical spectral changes were observed under 505 nm green-light excitation, which is beyond the absorption of the parent motor. Although explicit energy-transfer efficiency was not quantified, theoretical and experimental analyses indicated that synergistic intramolecular coupling enables population of the motor's S_1_ state following BODIPY excitation, thus achieving visible-light operation.

### 3.2. Energy transfer from nanomaterials

Beyond molecular chromophores, a variety of nanomaterials can serve as external light-harvesting platforms that convert NIR or visible excitation into the wavelengths required to drive rotary motion.

#### 3.2.1. Upconversion nanoparticles

In 2025, the Feringa group developed a general and efficient indirect strategy for powering molecular motors with NIR light by employing lanthanide-doped upconversion nanoparticles (UCNPs) as internal light transducers (Fig. 4b).^72^ Conventional two-photon activation of molecular motors typically requires high-intensity femtosecond lasers together with extensive π-system engineering, which greatly limits practical applicability, particularly in condensed phases and biological environments. In contrast, the UCNPs used in this study absorb low-energy 980 nm irradiation and emit narrow-band UV/visible light in the 340–470 nm region, which overlaps well with the absorption bands of many overcrowded-alkene-based molecular motors. Owing to their long emission lifetimes, high photostability, and low cytotoxicity, these nanoparticles function as efficient nanoscale transducers that convert biologically compatible NIR light into higher-energy photons capable of driving motor photoisomerisation.

The study examined several representative motor structures and established that a highly photoefficient first-generation motor 22 (*λ*_max_ ≈ 365 nm, *t*_1/2_ ≈ 29 h at 20 °C) could be effectively operated under continuous-wave 980 nm radiation in the presence of UCNPs. Radiative energy transfer from the nanoparticle emission promoted the usual photoisomerisation, yielding a PSD comparable to that obtained under direct UV irradiation. The process required only moderate light intensity and proceeded smoothly in air, and it is universally applicable: other second-generation motors and overcrowded alkene-derived bistable switches showed similar efficiency,^72^ demonstrating that NIR activation can be achieved without any structural modification of the motor itself.

Subsequently, this UCNP-assisted strategy was further extended to responsive liquid-crystal (LC) systems using intrinsically chiral formylated molecular motors as photochemical dopants.^73^ As a result of the high photoefficiency and multistate dynamic chirality of the motor, reversible quadruple helicity inversion of cholesteric LC superstructures was achieved together with exceptionally large HTP modulation. By incorporating NaYF_4_-based upconversion nanoparticles (UCNPs), they further demonstrated the first example of reversible NIR-light-induced helicity inversion of LC phases under 980 nm irradiation.

#### 3.2.2. Metal–organic framework

The Feringa group introduced a fundamentally different approach based on noncovalent energy transfer within a photoactive metal–organic framework (MOF) in 2020 (33, Fig. 4c).^74^ In this system, Pd–porphyrin linkers acted as light-harvesting antennas that absorbed visible photons and relayed their triplet energy to embedded overcrowded-alkene-based motors serving as structural pillars. This spatially separated yet electronically coupled arrangement enabled the motors to undergo reversible photoisomerisation and THI upon green-light irradiation (*λ*_irr_ = 530 nm), even in the crystalline solid. Remarkably, the rotational dynamics, that is, the rate of thermal helix inversion, within the MOF porous material were nearly identical to those in solution (Δ^‡^*G* ≈ 88 kJ mol^−1^), demonstrating that efficient triplet–triplet energy transfer (TTET) can drive unidirectional motion without the need for direct covalent conjugation.

#### 3.2.3. Quantum dots

In 2025, Liu *et al.* introduced another strategy by employing quantum dots (QDs) as external light-harvesting sensitizers to drive molecular motors across the entire visible spectrum (Fig. 4d).^75^ This system relies on noncovalent, two-step triplet energy transfer (TET) relay in a mixed colloidal environment. Upon visible-light excitation, CdSe QDs of tunable size absorb photons from green to red light and transfer their triplet energy first to an anthracene mediator (9-anthracenecarboxylic acid), which subsequently relays the energy to the motor, initiating its photoisomerisation and THI processes. This elegant “cocktail” approach enables efficient motor rotation even under weak LED irradiation, without any structural modification of the motor itself.

Together, these examples demonstrate that long-wavelength activation does not need to be engineered into the motor scaffold itself. Instead, light-harvesting chromophores and nanomaterials can function as external transducers that bridge the mismatch between benign excitation wavelengths and the intrinsic absorption profile of overcrowded-alkene motors. A major advantage of these indirect strategies over direct molecular redesign is that they bypass the need to construct entirely new motor structures, avoiding lengthy synthetic routes and extensive structural optimization. Instead, existing motors can be activated with visible or NIR light simply by pairing them with an appropriate external light-harvesting platform. The challenge, however, lies in judiciously selecting sensitizers or nanomaterials whose emission profiles, excited-state lifetimes, and energy-transfer pathways are well matched to the motor's photochemical requirements. In this sense, indirect activation offers efficiency and modularity, but demands a deeper understanding of photophysical properties, especially regarding compatibility to achieve optimal performance.

## 4. Applications

Over the past two decades, molecular motors have been applied in diverse contexts—including integration into self-assembled systems, biological environments, functional materials, catalysts, surface modifications, and molecular therapeutics—demonstrating how light-driven rotary motion can regulate structure, dynamics, and function across multiple length scales.^76^ However, implementations utilizing visible or near-infrared (NIR) light as the driving source remain scarce, despite the clear advantages of low-energy excitation for soft materials and biological media where photostability and biocompatibility are critical.

Here we will introduce representative examples of visible-light- and NIR-light-driven molecular motors that bridge the gap between photochemical motion and functional performance. Although most systems remain at a proof-of-concept stage, they reveal the feasibility of translating molecular rotation into tangible outputs. Among these, the series of studies by the Tour group provides a particularly illustrative case, demonstrating how systematic molecular engineering can progressively shift the operational wavelength from the UV to the visible and NIR regions.

The Tour group pioneered the use of molecular motors to trigger biological responses by integrating Feringa-type rotary units into functional molecular nanomachines (Fig. 5a and b).^77–80^ In their early studies, these motors, which are in principle capable of operating at ultrafast frequencies (2–3 MHz) under UV excitation, were shown to induce rapid cell death upon light irradiation in biological environments.^78,80^ Subsequent derivatives were modified with fluorescent tags for imaging, nonspecific hydrophilic groups to improve membrane association, or peptide ligands to achieve tumour selectivity. These structural variations did not alter the photochemical mechanism but enhanced biological compatibility and specificity.

**Fig. 5:**
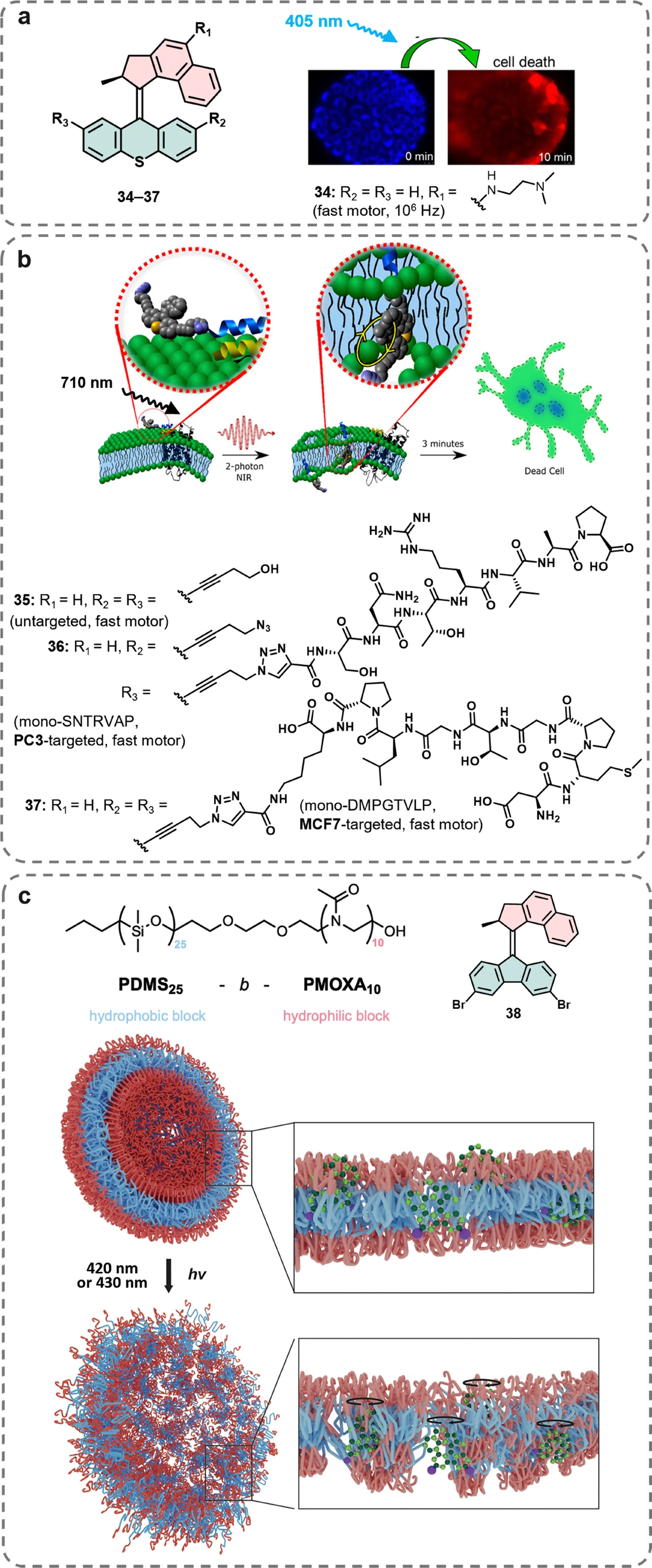
Application examples of visible or NIR-light-driven molecular motors in biology. (a) Cell death caused by molecular motor 34 under microscope excitation at 405 nm. Adapted with permission from ref. 77. Copyright 2020, American Chemical Society. (b) Schematic of molecular motors (35–37) selectively binding to the cell membrane through peptide-mediated recognition and subsequently inducing membrane disruption upon NIR two-photon excitation (710 nm), ultimately leading to targeted cell death. Adapted with permission from ref. 79. Copyright 2019, American Chemical Society. (c) Chemical structure of the PDMS_25_-*b*-PMOXA_10_ diblock copolymer and molecular motor 38 (top). Schematic representation of the vesicular system and magnification region of the multicomponent motor-block copolymer assembly in the bilayer of the delivery system (bottom). Adapted with permission from ref. 83. Copyright 2023, Proceedings of the National Academy of Sciences.

To minimize photodamage while preserving the biological effect, the Tour group subsequently employed visible-light (405 nm) irradiation, demonstrating that the same overcrowded-alkene framework could function efficiently under lower-energy excitation (Fig. 5a).^77^ Despite the reduced photon energy, blue-light activation produced comparable rates of cell death, accompanied by characteristic morphological changes such as membrane blebbing and cytoplasmic degradation, while exhibiting markedly lower phototoxicity toward the surrounding tissue.

Meanwhile, two-photon excitation at 710 nm enabled non-invasive operation in live-cell environments, where peptide-functionalized motors selectively targeted cancer cell lines such as PC-3 and MCF-7 and induced rapid membrane rupture within minutes of irradiation (Fig. 5b).^79^ Together, these studies provide elegant and important demonstrations of how progressive chemical functionalization of overcrowded-alkene motors has transformed them from model systems into biocompatible, light-activated molecular tools capable of controlled membrane disruption and targeted cell death. Although the mechanism underlying these observations is still subject of investigation, and more studies are needed to elucidate the factors contributing to the biological effects of these artificial molecular machines, these systems show remarkable potential.^81,82^

Another biological example was later reported by Feringa and Palivan in 2023, who integrated visible-light-driven molecular motors into a polymersome-based drug delivery system (Fig. 5c).^83^ In their study, a hydrophobic second-generation overcrowded-alkene motor was embedded into a PDMS–PMOXA diblock copolymer polymersome, forming a light-responsive nanocarrier system. Upon low-power blue-light irradiation (*λ*_irr_ = 420 or 430 nm, 6.9 mW), the unidirectional rotary motion of the motor generated sufficient mechanical stress to induce membrane rupture and guest release without chemical degradation or heating effects. The system exhibited on–off controllability over multiple irradiation cycles, achieving up to 75% release efficiency with only 1–25 mol% motor loading.

Importantly, this visible-light-responsive nanocarrier was capable of releasing an FDA-approved anticancer drug (pemetrexed) inside human lung carcinoma (A549) cells, showing comparable cytotoxicity to the free drug yet without inherent polymer or motor toxicity. These results provide the first proof-of-concept of a visible-light-driven molecular motor integrated into a polymeric drug-delivery platform, bridging the gap between molecular motion and practical nanomedicine.

In addition to biological applications, recent efforts have explored the integration of visible-light-responsive molecular motors into functional materials, aiming to translate molecular-scale rotation into controllable macroscopic changes in morphology, optics, and mechanics.

The Feringa group assembled a trimeric motor–metallacycle (14b, Fig. 2e; 2.3 metal-coordination) with heparin through multivalent electrostatic interactions to form a multicomponent, hierarchical network.^52^ SEM/TEM and EDX confirmed the mixed assemblies (Fig. 6a). Under 420 nm irradiation, the network reversibly reorganizes—from 3D cross-linked frameworks to lamellar aggregates (≈2 h) and then to linked/dispersed nanospheres (≈4 h)—and, after thermal relaxation in the dark (≈12 h), returns to its original morphology. These changes show that visible-light-induced isomerisation of the embedded motors modulates secondary aggregation without disrupting the primary metallacycle structure. Consistently, DLS records a size shift from 615 nm (network state) to 220 nm (small aggregates) under 420 nm, followed by recovery upon ageing, highlighting the motors as a photoresponsive supramolecular “glue” that dynamically cross-links and decross-links polymeric networks.

**Fig. 6:**
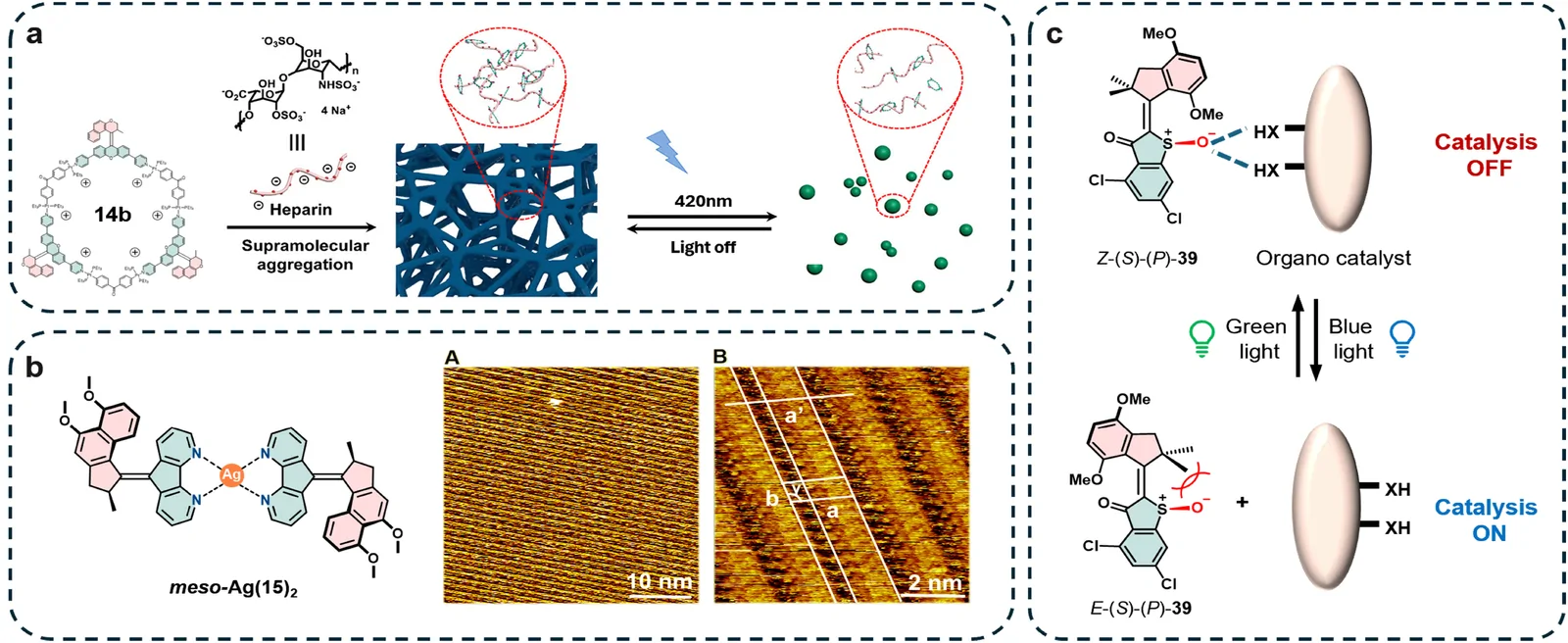
Illustrative applications of visible- or NIR-light-driven molecular motors in other fields. (a) Schematic representation of the manipulation of supramolecular aggregation of metallacycle 14b and heparin. Adapted with permission from ref. 52. Copyright 2020, American Chemical Society. (b) STM image of the self-assembled (A) overview and (B) zoomed-in structures of *meso*-Ag(15)_2_ on highly oriented pyrolytic graphite. Adapted with permission from ref. 53. Copyright 2021, the Royal Society of Chemistry. (c) Supramolecular integration of HTI molecular motor 39 to control organocatalysis.

Beyond these heparin-cross-linked networks, metal coordination has likewise been used to organize motors into a “two-wheel nanocar” that operates under visible light.^53,84^ In this design, two opposite enantiomers of motor (15) are bridged by a central pseudo-square-planar Ag(i) node to give the dimeric complex meso-Ag(15)_2_—a minimal “two-wheel” chassis in which both wheels co-rotate in the same direction to the external observer (Fig. 2f). The assembly proceeds in one-pot, and the structure was confirmed crystallographically; coordination contracts the diazafluorene stator, thereby changing the steric interactions between rotor and stator in the ‘*fjord’* region and tuning the rotation kinetics of the wheels. Due to the ligand's push–pull electronics and ^3^ILCT sensitization, this nanocar is addressable with visible light up to 500 nm. Notably, the device is chemically reconfigurable: adding pyridine competes for complexation of Ag(i), resulting in the disassembly of the *meso*-Ag(15)_2_ back to free 15. On surfaces, STM revealed well-ordered, tilted layered arrays on the HOPG/octanol interface that mirror the crystal packing, suggesting a route toward single-molecule manipulation on interfaces (Fig. 6b).

Visible-light-driven motors have also been embedded in soft gels and polymer films, enabling operation in condensed phases. In a representative study, Zhang *et al.* designed benzofuranone-based rotary motors with red-shifted absorption (addressable up to 550 nm).^62^ They demonstrated robust performance by (i) doping the motors into a supramolecular gel, where photoisomerisation resulted in photostationary states comparable to those in solution, and (ii) incorporating them into polyvinylidene fluoride hexafluoropropylene (PVDF-HFP) films (motor : polymer ≈ 3 : 100 mol ratio), where UV-vis spectra showed clean isosbestic points and reversible formation of metastable isomers, confirming the photoisomerisation in the polymer matrix under visible light. Collectively, these results outline a practical route from solution studies to processable motorized materials.

Toward motor-based adaptive chiral catalysts,^85^ an elegant functional demonstration of visible-light-driven molecular motion was reported by the Dube group,^24^ who achieved supramolecular relay control of organocatalysis using a hemithioindigo (HTI) motor (Fig. 6c). In their design, the motor forms hydrogen bonds with common H-bonding organocatalysts such as Schreiner's thiourea and squaramide derivatives. Light-induced isomerisation toggles the motor between a high-affinity C state and a low-affinity A state, thereby switching the catalyst between inactive and active forms without any covalent modification. The change in association constant exceeds one order of magnitude (from 2300 to 200 L mol^−1^), directly translating into reversible modulation of reaction rates in a model Michael addition reaction. The system operates cleanly under visible-light irradiation (450–520 nm) and benefits from the motor's high thermal bistability (Δ^‡^*G* > 134 kJ mol^−1^), allowing precise kinetic control over long timescales. This work again elegantly exemplifies how visible-light-driven rotary motors can be harnessed as molecular regulators, converting photochemical motion into dynamic chemical function.

Another emerging application of visible-light-responsive molecular motors is molecular solar thermal (MOST) energy storage.^86^ Recently, Dube and co-workers demonstrated that a supercharged HTI motor could be directly charged by sunlight, allowing efficient population of a high-energy metastable state at low temperature (16d, Fig. 3a).^54^ Although currently limited to proof-of-concept operation under cryogenic conditions, this work represents the first demonstration of a molecular motor in a MOST system and highlights the broader opportunities created by extending motor absorption toward the visible and sunlight regions.

## 5. Outlook and conclusion

Over the past two decades, the field of light-driven molecular motors has advanced through the persistent efforts of chemists who have continuously refined the relationship between molecular structure, electronic design, and photochemically driven motion. This review has summarized the diverse strategies developed to push the operational wavelengths from the ultraviolet to the visible and near-infrared regions, a transformation that represents not only a technical achievement but also a conceptual shift toward more practical and versatile molecular machines.

These strategies illustrate that visible and NIR light activation can be achieved either by tuning the intrinsic electronic structure of the motor or by harnessing external light-harvesting components, offering a versatile foundation for practical applications. To translate these design features into reliable wavelength control, however, several key challenges must still be addressed:

(i) Expanding the operational wavelength range

Although numerous molecular design strategies have been proposed to red-shift the absorption and irradiation wavelengths of light-driven molecular motors, most reported systems still operate within a relatively narrow window, typically between 400 and 500 nm. Motors that can be efficiently excited at longer wavelengths—particularly toward the deep-visible, red, or NIR regions—remain scarce. This imbalance highlights the need for new molecular architectures and mechanistic approaches, or for extending current strategies in more versatile ways, to create a broader family of motor designs and structures capable of covering the full UV-Vis-NIR spectrum. Achieving such spectral diversity would not only expand the operational flexibility of molecular motors but also enable wavelength-tailored functions across different materials and environments.

(ii) Improving photochemical efficiency and energy utilization

Most visible and NIR-light-responsive molecular motors still suffer from poor photoefficiency, reflected in low QYs of isomerisation and poor PSDs. Although ultrafast spectroscopy and quantum-chemical simulations have advanced rapidly, the excited-state dynamics of molecular motors remain far from fully elucidated. Many key events, from the initial Franck–Condon relaxation to the ultrafast structural rearrangements that lead to productive photoisomerisation, occur on femtosecond to picosecond timescales and therefore cannot be captured by conventional spectroscopic techniques. As a result, we still often lack a clear picture of how molecular structure governs excited-state behaviour, why certain motors exhibit inefficient energy conversion, and how these processes can be rationally optimized.

Addressing these questions will require the combined use of advanced ultrafast spectroscopies, high-level quantum chemical calculations, and structure–dynamics correlation studies. Resolving these fundamental mechanistic details is essential for improving light-harvesting capability, enhancing photoefficiency, and enabling the reliable operation of molecular motors under practical conditions.

(iii) Orthogonal and multi-input control of molecular motion

Coupling or decoupling molecular motions represents an important direction for increasing the functional complexity of visible and NIR-light-driven molecular motors. Future systems are expected to move beyond isolated molecular designs toward more complex architectures, in which motor units are embedded within multifunctional environments and can operate either cooperatively or independently.

Orthogonal control through multiple external stimuli, including light of different wavelengths as well as thermal, redox, or chemical inputs, enables selective activation of individual motor units at the system level.^87–89^ Such multi-stimuli responsiveness underpins multifunctionality, while the ability to selectively and predictably control individual components establishes the basis for programmability.

(iv) Polyfunctional molecular motor designs

To achieve advanced applications in complex scenarios, enhanced functional complexity, *i.e.*, polyfunctional molecular motor designs with high photoefficiency, is a prerequisite. These systems, combining efficient unidirectional motion with custom-tailored auxiliary functionalities, are a key step towards molecular machinery.

In this regard, modular and synthetically facile design strategies are particularly valuable and timely needed. Typically, stepwise installation of functional groups onto a single motor scaffold *via* late-stage functionalization represents a prevalent yet time-intensive and synthetically challenging approach. Accordingly, developing innovative methodologies to access targeted, tunable molecular motors that integrate high functional versatility with excellent photoefficiency is critically important. Such advances enable the systematic fabrication of high-potential motor frameworks while retaining core photochemical properties, thereby facilitating the elucidation of structure–property relationships and guiding the rational design of sophisticated and programmable next-generation molecular machinery.

Although significant challenges remain in achieving precise and programmable control over molecular motion, these advances collectively open numerous opportunities to achieve responsive functions and translate visible- and NIR-light-driven motor dynamics into functional materials. Many promising directions are still in their infancy, and substantial room exists to explore how such motors can be synchronized and operate cooperatively within complex architectures:

(i) Molecular motors for applications in materials science.

Recent integration of light-driven molecular motors into polymeric, supramolecular, crystalline systems and responsive surfaces has opened new opportunities for modulating macroscopic mechanical and optical properties with light. In soft materials such as polymer networks and gels, the collective motion of embedded motors can induce contraction, expansion, bending, or stress release, enabling reversible photomechanical actuation.^90,91^ In addition, these systems can also support light-to-energy conversion and storage, as exemplified by molecular solar thermal (MOST) systems, in which absorbed photon energy is stored in metastable isomers and released on demand as heat.^92^

Beyond soft materials, incorporation of molecular motors into liquid-crystalline and crystalline matrices enables cooperative reorientation and reversible phase transitions, providing dynamic control over anisotropy, color, and refractive properties.^93^ These concepts have been further extended to framework materials, such as metal–organic frameworks (MOFs), covalent organic frameworks (COFs), and porous aromatic frameworks (PAFs), enabling collective and spatially organized molecular motion within rigid scaffolds.^74,88,94^ At a more functional level, the ability of visible-light- and NIR-light-driven motors to achieve spatially resolved activation deep within condensed materials enables patterned stress generation, chiral domain writing, and the development of functional devices such as soft robotic components, microfluidic valves, and reconfigurable optical systems.

Together, these advances demonstrate the potential of molecular motors to bridge length scales, from the molecular to the macroscopic level, coupling molecular-level photochemistry with dynamic macroscopic functions. It also highlights pathways to smart (self-cleaning, self-repair) surfaces and toward adaptive and energy-efficient photomechanical materials.

(ii) Molecular motors for application in catalysis

The intrinsic chirality, conformational asymmetry, and directional rotation of molecular motors also make them attractive platforms for dynamic and switchable catalysis.^4,24,95^ Upon light irradiation, these systems can modulate both steric and electronic environments, enabling temporal control over reactivity and selectivity.

Effective catalytic operation further requires well-defined PSDs across different rotational states, as well as sufficient structural robustness under repeated switching cycles, since both directly impact catalytic performance and reproducibility. In this regard, visible and NIR-light-driven motors are particularly advantageous, as long-wavelength excitation reduces photodamage and suppresses undesired side reactions, thereby improving operational stability over extended use.

(iii) Molecular motors for applications in biology

Molecular motors have emerged as promising tools for biomedical and therapeutic applications, especially in photopharmacology,^96–100^ functioning as photoactuated nanomechanical devices capable of converting light into localized mechanical work.^77,79,83^ At the molecular and cellular levels, their directional motion can induce conformational changes, mechanical tension, or local perturbation of biological structures, thereby influencing biochemical processes with subcellular precision. Motors embedded within liposomes, hydrogels, or membrane systems can regulate permeability or trigger site-specific drug release under optical stimulation, offering non-invasive routes for controlled delivery and activation. In addition, light-driven modulation of protein conformation, DNA binding, enzyme activity, or receptor signaling presents powerful opportunities for synthetic biology and dynamic regulation of living systems. A prominent example is our demonstration of stem cell differentiation controlled by light-driven motors assembled on surfaces.^101^

The extension of these systems into the visible and near-infrared regions brings decisive advantages for biological operation. The deeper penetration and low phototoxicity of long-wavelength light enable safe and efficient actuation in tissue environments that are inaccessible to UV light. The mild optical conditions of visible and NIR excitation further ensure *in vivo* compatibility, supporting the development of mechanotherapeutic agents that can perform targeted mechanical tasks inside biological systems under external light control.^98,100^

### 5.1. Future directions toward predictive molecular motor design

Despite the remarkable progress achieved in molecular motor design, the discovery of high-performance systems still relies heavily on empirical trial-and-error approaches. As molecular architectures become increasingly sophisticated and multifunctional, identifying optimal combinations of structural features through conventional synthetic screening alone is becoming progressively more challenging. Computational chemistry, high-throughput screening, machine learning, and AI-assisted molecular design are therefore expected to play increasingly important roles in accelerating the rational development of next-generation molecular motors.

Importantly, future design strategies should extend beyond optimizing isolated molecular structures to considering the environments in which molecular motors ultimately operate. Solvent polarity, viscosity, non-covalent interactions, molecular confinement, and surrounding materials can all profoundly influence excited-state relaxation, photoisomerisation quantum yields, thermal steps, and ultimately the overall efficiency of the motor cycle. Integrating molecular structure, photochemical performance, and operational environments into predictive design frameworks will not only facilitate the rational development of increasingly robust and programmable molecular motors but also accelerate their translation from proof-of-concept molecular systems toward practical molecular machinery.

Ultimately, the pursuit of visible- and near-infrared-light-driven molecular motors is about more than simply shifting excitation wavelengths toward longer wavelengths. It reflects a broader ambition to control molecular motion with light in a manner that is precise, efficient, and compatible with increasingly complex environments. Over the past two decades, molecular motors have evolved from elegant demonstrations of unidirectional motion into increasingly sophisticated systems capable of performing functions across molecular, material, and biological settings. Yet considerable opportunities remain before such motion can be programmed and harnessed with the reliability expected of truly functional molecular machinery. Continued advances in molecular design, mechanistic understanding, predictive tools, and system-level integration will progressively narrow this gap. As these boundaries are pushed further, visible and near-infrared light may provide a powerful means of bringing artificial molecular motion closer to the spatial precision, temporal control, and functional complexity that characterize molecular machinery in nature.

## Conflicts of interest

There are no conflicts to declare.

## Data Availability

No primary research results, software or code have been included and no new data were generated or analysed as part of this review.

## References

[cit1] McIlwain J. T. , An introduction to the biology of vision, Cambridge university press, Cambridge New York Melbourne, 1996

[cit2] Blankenship R. E. , Molecular mechanisms of photosynthesis, Wiley, Hoboken, 3rd edn, 2022

[cit3] Sancar A. (2003). Chem. Rev..

[cit4] García-López V., Liu D., Tour J. M. (2020). Chem. Rev..

[cit5] Sauvage J. (2017). Angew. Chem., Int. Ed..

[cit6] Stoddart J. F. (2017). Angew. Chem., Int. Ed..

[cit7] Coskun A., Banaszak M., Astumian R. D., Stoddart J. F., Grzybowski B. A. (2012). Chem. Soc. Rev..

[cit8] Balzani V., Credi A., Venturi M. (2009). Chem. Soc. Rev..

[cit9] Giuseppone N. (2012). Acc. Chem. Res..

[cit10] Kinbara K., Aida T. (2005). Chem. Rev..

[cit11] Feringa B. L., Jager W. F., De Lange B., Meijer E. W. (1991). J. Am. Chem. Soc..

[cit12] Koumura N., Zijlstra R. W. J., Van Delden R. A., Harada N., Feringa B. L. (1999). Nature.

[cit13] Klok M., Boyle N., Pryce M. T., Meetsma A., Browne W. R., Feringa B. L. (2008). J. Am. Chem. Soc..

[cit14] Koumura N., Geertsema E. M., Van Gelder M. B., Meetsma A., Feringa B. L. (2002). J. Am. Chem. Soc..

[cit15] Vicario J., Meetsma A., Feringa B. L. (2005). Chem. Commun..

[cit16] Kistemaker J. C. M., Štacko P., Visser J., Feringa B. L. (2015). Nat. Chem..

[cit17] Lehn J. (2006). Chem. – Eur. J..

[cit18] Greb L., Lehn J.-M. (2014). J. Am. Chem. Soc..

[cit19] Guentner M., Schildhauer M., Thumser S., Mayer P., Stephenson D., Mayer P. J., Dube H. (2015). Nat. Commun..

[cit20] Huber L. A., Hoffmann K., Thumser S., Böcher N., Mayer P., Dube H. (2017). Angew. Chem., Int. Ed..

[cit21] Wilcken R., Schildhauer M., Rott F., Huber L. A., Guentner M., Thumser S., Hoffmann K., Oesterling S., De Vivie-Riedle R., Riedle E., Dube H. (2018). J. Am. Chem. Soc..

[cit22] Gerwien A., Schildhauer M., Thumser S., Mayer P., Dube H. (2018). Nat. Commun..

[cit23] Gerwien A., Mayer P., Dube H. (2019). Nat. Commun..

[cit24] Grill K., Dube H. (2020). J. Am. Chem. Soc..

[cit25] Scaiano J. C. (2023). Chem. Soc. Rev..

[cit26] Pooler D. R. S., Lubbe A. S., Crespi S., Feringa B. L. (2021). Chem. Sci..

[cit27] Roy P., Sardjan A. S., Danowski W., Browne W. R., Feringa B. L., Meech S. R. (2023). J. Am. Chem. Soc..

[cit28] Astumian R. D. (2017). Chem. Sci..

[cit29] Filatov M., Olivucci M. (2014). J. Org. Chem..

[cit30] Astumian R. D. (2023). Matter.

[cit31] Klok M., Browne W. R., Feringa B. L. (2009). Phys. Chem. Chem. Phys..

[cit32] Schuurmans J. H. A., Lukas F., Tiwari P. C., Noël T. (2026). Acc. Chem. Res..

[cit33] Kim H. M., Cho B. R. (2015). Chem. Rev..

[cit34] Yousif E., Haddad R. (2013). SpringerPlus.

[cit35] Meinhardt M., Krebs R., Anders A., Heinrich U., Tronnier H. (2008). J. Biomed. Opt..

[cit36] Liu P., Mu X., Zhang X.-D., Ming D. (2020). Bioconjugate Chem..

[cit37] Van Leeuwen T., Pol J., Roke D., Wezenberg S. J., Feringa B. L. (2017). Org. Lett..

[cit38] Roke D., Feringa B. L., Wezenberg S. J. (2019). Helv. Chim. Acta.

[cit39] Roke D., Stuckhardt C., Danowski W., Wezenberg S. J., Feringa B. L. (2018). Angew. Chem., Int. Ed..

[cit40] Pfeifer L., Hoang N. V., Crespi S., Pshenichnikov M. S., Feringa B. L. (2022). Sci. Adv..

[cit41] Van Vliet S., Sheng J., Stindt C. N., Feringa B. L. (2024). Nat. Commun..

[cit42] Dang T., Zhang Z.-Y., Li T. (2024). J. Am. Chem. Soc..

[cit43] Volarić J., Szymanski W., Simeth N. A., Feringa B. L. (2021). Chem. Soc. Rev..

[cit44] Van Delden R. A., Koumura N., Schoevaars A., Meetsma A., Feringa B. L. (2003). Org. Biomol. Chem..

[cit45] Pollard M. M., Wesenhagen P. V., Pijper D., Feringa B. L. (2008). Org. Biomol. Chem..

[cit46] Pfeifer L., Scherübl M., Fellert M., Danowski W., Cheng J., Pol J., Feringa B. L. (2019). Chem. Sci..

[cit47] Pfeifer L., Crespi S., Van Der Meulen P., Kemmink J., Scheek R. M., Hilbers M. F., Buma W. J., Feringa B. L. (2022). Nat. Commun..

[cit48] Wezenberg S. J., Chen K., Feringa B. L. (2015). Angew. Chem., Int. Ed..

[cit49] Faulkner A., Van Leeuwen T., Feringa B. L., Wezenberg S. J. (2016). J. Am. Chem. Soc..

[cit50] Stindt C. N., Crespi S., Toyoda R., Hilbers M. F., Kemmink J., Van Der Meulen P., Buma W. J., Feringa B. L. (2023). Chem.

[cit51] Stindt C. N., Jo T., Steen J. D., Feringa B. L., Crespi S. (2025). J. Phys. Chem. A.

[cit52] Shi Z.-T., Hu Y.-X., Hu Z., Zhang Q., Chen S.-Y., Chen M., Yu J.-J., Yin G.-Q., Sun H., Xu L., Li X., Feringa B. L., Yang H.-B., Tian H., Qu D.-H. (2021). J. Am. Chem. Soc..

[cit53] Li M., Li S., Zhang K., Chi X., Zhou H., Xu H.-B., Zhang Y., Li Q., Wang D., Zeng M.-H. (2021). Nanoscale.

[cit54] Biswas P. K., Ozcelik A., Hartinger M., Groß N., Hampel F., Müller C., Dube H. (2026). Nat. Chem..

[cit55] Roke D., Sen M., Danowski W., Wezenberg S. J., Feringa B. L. (2019). J. Am. Chem. Soc..

[cit56] Pooler D. R. S., Pierron R., Crespi S., Costil R., Pfeifer L., Léonard J., Olivucci M., Feringa B. L. (2021). Chem. Sci..

[cit57] Pooler D. R. S., Doellerer D., Crespi S., Feringa B. L. (2022). Org. Chem. Front..

[cit58] Van Beek C. L. F., Feringa B. L. (2024). J. Am. Chem. Soc..

[cit59] Sheng J., Danowski W., Sardjan A. S., Hou J., Crespi S., Ryabchun A., Domínguez M. P., Jan Buma W., Browne W. R., Feringa B. L. (2024). Nat. Chem..

[cit60] Ren J., Pooler D. R. S., Guo H., Tu Y., Danowski W., Xu F., Feringa B. L., Sheng J. (2026). J. Am. Chem. Soc..

[cit61] Sheng J., Van Beek C. L. F., Stindt C. N., Danowski W., Jankowska J., Crespi S., Pooler D. R. S., Hilbers M. F., Buma W. J., Feringa B. L. (2025). Sci. Adv..

[cit62] Zhang C., Jiang Z., Qin Y., Fu Y., Li Q., Zhang Y., Zeng M.-H. (2022). Mater. Today Chem..

[cit63] Kuntze K., Pooler D. R. S., Di Donato M., Hilbers M. F., Van Der Meulen P., Buma W. J., Priimagi A., Feringa B. L., Crespi S. (2023). Chem. Sci..

[cit64] Wang J., Durbeej B. (2018). ChemistryOpen.

[cit65] Balashov S. P., Imasheva E. S., Boichenko V. A., Antón J., Wang J. M., Lanyi J. K. (2005). Science.

[cit66] Blankenship R. E. , Molecular mechanisms of photosynthesis, Blackwell Science, Oxford Malden, MA, 2002

[cit67] Masters B. R. (2014). Eur. Phys. J. H.

[cit68] Dexter D. L. (1953). J. Chem. Phys..

[cit69] Cnossen A., Hou L., Pollard M. M., Wesenhagen P. V., Browne W. R., Feringa B. L. (2012). J. Am. Chem. Soc..

[cit70] Pfeifer L., Hoang N. V., Scherübl M., Pshenichnikov M. S., Feringa B. L. (2020). Sci. Adv..

[cit71] Toyoda R., Hoang N. V., Moghaddam K. G., Crespi S., Pooler D. R. S., Faraji S., Pshenichnikov M. S., Feringa B. L. (2022). Nat. Commun..

[cit72] Sheng J., Fu Y., Wu K., Freese T., Zhang H., Feringa B. L. (2025). J. Am. Chem. Soc..

[cit73] Sheng J., Ryabchun A., Long G., Feringa B. L. (2026). Adv. Mater..

[cit74] Danowski W., Castiglioni F., Sardjan A. S., Krause S., Pfeifer L., Roke D., Comotti A., Browne W. R., Feringa B. L. (2020). J. Am. Chem. Soc..

[cit75] Liu J., Zhang S., Xi L., Liu J., He Y., Wang R., Qu D.-H., Feringa B. L., Liu T., Hou L. (2025). J. Am. Chem. Soc..

[cit76] Foy J. T., Li Q., Goujon A., Colard-Itté J.-R., Fuks G., Moulin E., Schiffmann O., Dattler D., Funeriu D. P., Giuseppone N. (2017). Nat. Nanotechnol..

[cit77] Ayala Orozco C., Liu D., Li Y., Alemany L. B., Pal R., Krishnan S., Tour J. M. (2020). ACS Appl. Mater. Interfaces.

[cit78] García-López V., Chiang P.-T., Chen F., Ruan G., Martí A. A., Kolomeisky A. B., Wang G., Tour J. M. (2015). Nano Lett..

[cit79] Liu D., García-López V., Gunasekera R. S., Greer Nilewski L., Alemany L. B., Aliyan A., Jin T., Wang G., Tour J. M., Pal R. (2019). ACS Nano.

[cit80] García-López V., Chen F., Nilewski L. G., Duret G., Aliyan A., Kolomeisky A. B., Robinson J. T., Wang G., Pal R., Tour J. M. (2017). Nature.

[cit81] Firsov A. M., Pfeffermann J., Benditkis A. S., Rokitskaya T. I., Kozlov A. S., Kotova E. A., Krasnovsky A. A., Pohl P., Antonenko Y. N. (2023). J. Photochem. Photobiol., B.

[cit82] Guinart A., Qutbuddin Y., Schwille P., Feringa B. L. (2025). Nat. Rev. Chem..

[cit83] Guinart A., Korpidou M., Doellerer D., Pacella G., Stuart M. C. A., Dinu I. A., Portale G., Palivan C., Feringa B. L. (2023). Proc. Natl. Acad. Sci. U. S. A..

[cit84] Kudernac T., Ruangsupapichat N., Parschau M., Maciá B., Katsonis N., Harutyunyan S. R., Ernst K.-H., Feringa B. L. (2011). Nature.

[cit85] Wang J., Feringa B. L. (2011). Science.

[cit86] Wang Z., Hölzel H., Moth-Poulsen K. (2022). Chem. Soc. Rev..

[cit87] Zhu G., Sun F., Ji Y., Hu H., Yang M., Zhang Y., Deng X., Zheng Y., Wei C., Wang D. (2025). Chem. Soc. Rev..

[cit88] Sheng J., Perego J., Bracco S., Cieciórski P., Danowski W., Comotti A., Feringa B. L. (2024). Angew. Chem., Int. Ed..

[cit89] Köttner L., Dube H. (2024). Angew. Chem., Int. Ed..

[cit90] Foy J. T., Li Q., Goujon A., Colard-Itté J.-R., Fuks G., Moulin E., Schiffmann O., Dattler D., Funeriu D. P., Giuseppone N. (2017). Nat. Nanotechnol..

[cit91] Li Q., Fuks G., Moulin E., Maaloum M., Rawiso M., Kulic I., Foy J. T., Giuseppone N. (2015). Nat. Nanotechnol..

[cit92] Wang Z., Hölzel H., Moth-Poulsen K. (2022). Chem. Soc. Rev..

[cit93] Wang Y., Li Q. (2012). Adv. Mater..

[cit94] Sheng J., Perego J., Bracco S., Cieciórski P., Danowski W., Comotti A., Feringa B. L. (2026). Proc. Natl. Acad. Sci. U. S. A..

[cit95] Sheng J., Pooler D. R. S., Feringa B. L. (2023). Chem. Soc. Rev..

[cit96] Velema W. A., Szymanski W., Feringa B. L. (2014). J. Am. Chem. Soc..

[cit97] Broichhagen J., Frank J. A., Trauner D. (2015). Acc. Chem. Res..

[cit98] Lerch M. M., Hansen M. J., van Dam G. M., Szymanski W., Feringa B. L. (2016). Angew. Chem., Int. Ed..

[cit99] Welleman I. M., Hoorens M. W. H., Feringa B. L., Boersma H. H., Szymański W. (2020). Chem. Sci..

[cit100] Kobauri P., Dekker F. J., Szymanski W., Feringa B. L. (2023). Angew. Chem., Int. Ed..

[cit101] Zhou Q., Chen J., Luan Y., Vainikka P. A., Thallmair S., Marrink S. J., Feringa B. L., Van Rijn P. (2020). Sci. Adv..

